# Adsorption of some metal ions from wastewater using a novel composite of recycled chitosan and hydroxyapatite

**DOI:** 10.1038/s41598-026-64119-1

**Published:** 2026-08-05

**Authors:** Mohamed Abd Elghany, Mahmoud S. A. Shahin, Mohamed M. Elsenety, Elhassan A. Allam, Rabie Saad Farag

**Affiliations:** 1 Chemistry Department, Faculty of Science, Al-Azhar University, P.O. Box 11884, Cairo, Egypt; 2 Main Laboratories, Egyptian Armed Forces, Cairo, Egypt; 3 Nuclear Power Plants Authority (NPPA), P.O. Box 11381, Cairo, Egypt

**Keywords:** Adsorption, Hydroxyapatite, Chitosan, Metal ions, Nanocomposite, (HAp@CT) Wastewater, Chemistry, Environmental sciences, Materials science

## Abstract

In this study, an environmentally sustainable nanocomposite of recycled chitosan (CT) from shrimp waste and hydroxyapatite (HAp) was successfully synthesized and characterized. The adsorption performance of the prepared (HAp@CT) composite and its pristine components toward (Mn^2+^), (Fe^3+^), and (Pb^2+^) ions from aqueous solutions were systematically investigated. The structural and physicochemical properties were analyzed using Fourier Transform Infrared Spectroscopy (FT-IR), X-ray Diffraction (XRD), and Thermogravimetric Analysis (TGA) to confirm the functional group interactions, crystalline structure, and thermal stability. Transmission Electron Microscopy (TEM) revealed nanoscale CT particles (~ 5.31 nm) and highly crystalline HAp with well-defined lattice fringes. The measured interplanar spacings (1.603 and 1.714 nm) correspond to characteristic planes of the hexagonal HAp crystal system, confirming its ordered atomic arrangement. The batch adsorption was studied by optimizing the adsorption parameters. The maximum adsorption efficiency, were 160.20, 172.70, and 229.02 mg/g for Mn^2+^ using HAp, CT, and HAp@CT, respectively, for Pb^2+^ were 216.23, 201.28, and 179.33 mg/g, while for Fe^3+^ were 190.02, 200.31, and 206.92 mg/g, respectively, these values were obtained at 12.0 mg/L of initial concentration, 0.05 g/L of adsorbent dose, 120 min of contact time, and pH = 5.0–6.0, respectively. Overall, the results demonstrate that the HAp@CT nanocomposite exhibits competitive adsorption performance, particularly for Mn^2+^ and Fe^3+^ ions, while maintaining enhanced thermal stability inherited from the HAp component. These findings highlight the potential of sustainable biopolymer–inorganic hybrid nanocomposites for environmentally friendly water treatment applications and provide a foundation for further optimization and scale-up studies.

## Introduction

Recently, numerous studies have focused on water and wastewater treatment for the removal of various contaminants, particularly heavy metals. These metals pose significant environmental concerns due to their toxicity, non-biodegradability, and potential for bioaccumulation in living organisms^1^. Water contamination by hazardous substances, especially heavy metals has become one of the most critical environmental challenges in recent decades. Industrial and agricultural effluents contaminated with toxic metals pose severe risks to ecosystems and human health because of their persistence and bioaccumulation potential^2^. The development of a sustainable and low-cost adsorbent material has therefore attracted global attention has been widely explored to mitigate these contaminants^3^. Adsorption using low-cost and locally available materials remains one of the most promising solutions due to its simplicity, economic feasibility, and potential for resource recovery^4^. Toxic metals such as Pb^2+^, Cd^2+^, Hg^2+^, and Cr^3+^ are non-biodegradable and pose serious environmental and health risks, which has encouraged the development of recyclable HAp-based nanosorbents for water purification^5,6^.

A wide range of materials, including nanocomposites, nano metal oxides, nano-polymers, and metal–organic frameworks (MOFs), have been explored for this purpose. Nanocomposites generally provide enhanced adsorption performance through synergistic effects, whereas nano metal oxides exhibit high adsorption capacity but may suffer from aggregation. Nano-polymers offer abundant functional groups for metal binding, while MOFs possess exceptionally high surface areas but often require relatively complex synthesis and higher production costs. Therefore, the selection of an appropriate adsorbent depends on the targeted application, performance requirements, and economic considerations^7,8^.

HAp is a calcium phosphate with a composition similar to natural bone, has gained significant attention for environmental remediation due to its high ion-exchange capacity, biocompatibility, and ease of functionalization^9,10^. These properties make HAp an effective adsorbent for removing heavy metals pollutants from wastewater. Recent studies have shown that HAp nanocomposites exhibit enhanced adsorption performance when combined with organic or inorganic materials^11,12^. Various HAp-based composites have been developed for pollutant removal. For example, CT–HAp composites, manganese ferrite–HAp nanocomposites, and HAp/manganese dioxide systems have demonstrated for removal of heavy metals from aqueous solutions^13–16^. Incorporating natural polymers such as CT can further enhance adsorption capacity due to the presence of amino and hydroxyl functional groups^17,18^. Recent advances in nanotechnology have also promoted the development of nanocomposite adsorbents with high surface area and tunable structures for efficient wastewater treatment^19,20^. CT-based nanocomposites, such as magnetic CT/halloysite@ZnγFe₃O₄, have shown enhanced ability to remove multiple metal ions including Cr^3+^, Fe^3+^, and Mn^2+^. Many studies highlight CT and its composites as effective adsorbents for heavy metals^21,22^. Additionally, converting waste materials such as fish and shrimp shells into HAp/CT composites offers an environmentally friendly approach for large-scale applications^23,24^. The performance of HAp nanocomposites strongly depends on synthesis methods and morphology; for instance, porous HAp sheets prepared by non-aqueous precipitation exhibit improved Pb^2+^ removal efficiency^25^.

In this study, low-cost biowastes were valorized into high-value nanomaterials for the treatment of aqueous solutions contaminated with heavy metals. HAp was synthesized from fish bones, a natural and abundant source of calcium phosphate, while CT was obtained from shrimp shells via a deacetylation process. The two materials were subsequently integrated to fabricate HAp@CT nanocomposite with enhanced adsorption performance. This design combines the high ion-exchange capacity of HAp with the biocompatibility and abundant functional groups of CT, leading to improved adsorption efficiency. The developed approach not only converts biological waste into eco-friendly adsorbents but also contributes to sustainable waste management and environmental protection. The prepared nanosorbents (HAp, CT, and HAp@CT) were systematically characterized and evaluated for the removal of Mn^2+^, Fe^3+^, and Pb^2+^ ions from aqueous solutions. Adsorption performance and kinetic behavior were investigated, revealing the promising potential of the synthesized nanocomposite as an efficient adsorbent for heavy-metal remediation in water treatment applications. Finally, the proposed green synthesis approach demonstrates good potential for scalability and cost-effectiveness due to the use of abundant and inexpensive biowaste materials, including shrimp shells and Tilapia fish bones, as natural sources for chitosan and hydroxyapatite, respectively. The preparation procedures involve simple chemical treatments and conventional laboratory equipment without requiring sophisticated instruments or high-cost reagents. In addition, the recycling of seafood waste can control the environmental pollution and contributes to sustainable waste management. These advantages make the developed HAp@CT nanocomposite a promising low-cost and environmentally friendly material for large-scale wastewater treatment applications.

## Experimental

### Materials and instrumentation

Table 1, and 2 summarize the chemicals (analytical reagent grade, used without further purification) and the instruments employed in this study. All glassware used in the experiments was cleaned with 15% nitric acid, thoroughly rinsed with double-distilled water, and dried in an oven prior to use.

**Table 1:** Instrumentations used in the preparation.

Instrument name	Model	Data	Conditions	Technique
FT-IR	BRUKER Tensor 37	FT-IR spectrum	400–4000 cm^− 1^	Using KBr pellets
TGA-7 thermobalance	A Perkin-Elmer	Thermogram	Temperature heating (25–800 °C)	Pure nitrogen atmosphere, flow rate = 40 mL /min, heating rate = 10 °C/min and sample mass in the range of 5.0–6.0 mg
XRD	Shimadzu lab x 6100, Kyoto, Japan	XRD spectrum	40 kV, 30 mA, λ = 1 Å, 2θ from 10 to 80, recording steps of the diffraction data of 0.02°, at a time of 0.6 s, at room temperature (25 °C).	X-ray diffractometer, using target Cu-Kα
SEM	JSM-lT200, JEOL Ltd	SEM images	Imaging mode	Sputtering coating (JEOL-JFC-1100E)

**Table 2:** The chemical’s purity and the specifications of the used materials.

Chemical name	MF	FW (g/mol)	Company
Tripolyphosphate	Na_5_P_3_O_10_.	367.86	Sigma Aldrich, USA
Acetic acid	CH_3_CO_2_H	60.05	Elnasser chem. company
Sodium hydroxide	NaOH	39.997	Sigma Aldrich-Germany
Hydrochloric acid	HCl	36.46	Merck, Germany
Ferrous sulfate heptahydrate	FeSO_4_·7H_2_O	278.02	Sigma Aldrich
Manganese chloride hydrate	MnCl_2_·4H_2_O	197.90	
Absolute ethanol	C_2_H_6_O	46.07	Adwic, El-nasr pharm. chem co
Nitric acid (HNO_3_)	HNO_3_	63.013	Adwic, El-nasr pharm. chem co
Shrimp waste	[C_6_H_11_NO_4_]_n_	161.16	Commercial
Tilapia fish	Ca_5_(PO_4_)_3_OH	502.3	Commercial

### Methods

#### Preparation of chitosan nanoparticles

Shrimp waste collected from the local region was washed five to seven times with water, and soap, dried under open-air sunlight conditions, and crushed into fine particles. The resulting powder was converted into chitosan through sequential demineralization, deproteinization, washing/neutralization, and deacetylation steps. Demineralization and deproteinization were carried out to remove calcium carbonate and disrupt the chemical interactions between chitin and proteins using an acid–base treatment, in which the powder was treated separately with 1.0 M HCl (1:10 w/v) and 1.0 M NaOH (1:10 w/v) at room temperature for 24 h each^26^. After filtration, the residue was washed 3–5 times with deionized water (DI) until a neutral pH was achieved. During demineralization, calcium carbonate was converted into water-soluble calcium salts with the evolution of carbon dioxide^27^. The demineralized chitin was subsequently converted into chitosan by deacetylation through treatment with 60.0% sodium hydroxide solution under boiling conditions for 2.0 h. The alkaline solution was then removed, and the product was repeatedly washed with DI until neutral pH was reached, after which the obtained CT was dried at room temperature and stored for further use^28^. In this work, 0.50 g of the prepared CT was dissolved in 100 mL of 1% (v/v) acetic acid solution. The pH was adjusted to 4.60–4.80 using 1.0 M NaOH. CT nanoparticles were synthesized by adding 3.0 mL of the CT solution dropwise to 1.0 mL of 0.250% (w/v) aqueous TPP solution under vigorous magnetic stirring (CT: TPP ratio = 3:1). The nanoparticles formed spontaneously and were collected by centrifugation at 9000 × g for 20 min^28^. The obtained pellets were washed thoroughly with DI water to remove residual NaOH and then dried at 40 °C for 24 h before further use or analysis.

#### Preparation of natural nano-hydroxyapatite from fish bone

Natural nano-hydroxyapatite was prepared from Tilapia fish bones. 5.0 kg of fresh Tilapia were purchased, and all organic tissues, and adhering meat were carefully removed from the bones by thorough cleaning. The bones were repeatedly washed with DI and boiled in water for 2.0 h per day for three consecutive days to ensure the removal of residual organic matter. The cleaned bones were then dried at room temperature for 24 h. Subsequently, the dried bones were calcined in a muffle furnace at 700 °C for 1 h to remove any remaining organic components and to promote the crystallization of the HAp phase. After cooling to room temperature, the calcined bones were ground using an agate mortar to obtain a fine powder. The resulting nano-HAp powder was then subjected to physicochemical characterization^29^.

#### Preparation of HAp@CT nanocomposite

For the preparation of the HAp@CT nanocomposite, CT (3.5 g) was dissolved in 100 mL of 3% (v/v) acetic acid under magnetic stirring for 24 h at 30 °C. Subsequently, 0.50 g of HAp powder (corresponding to 10 wt% relative to CT) was added to the CT solution, and the mixture was stirred for an additional 24 h at 30 °C to ensure homogeneous dispersion. A sodium hydroxide solution was then added dropwise until the pH of the reaction medium reached 9.5, promoting the formation of the HAp@CT nanocomposite. The obtained product was washed 3–5 times with DI until neutral pH was reached and then dried in an oven at 60 °C until constant weight was achieved^30^.

### Adsorption of Mn^2+^, Fe^3+^, and Pb^2+^ from aqueous solution onto HAp@CT nanosorbent

Adsorption experiments were carried out to determine the optimal conditions for the removal of Mn^2+^, Fe^3+^, and Pb^2+^ ions from aqueous solutions using the HAp@CT nanosorbent. The experiments were performed using a mechanical shaker operating at 150 rpm to ensure effective mixing between the adsorbent and the metal ion solutions. The main parameters investigated included solution pH, contact time, and adsorbent dosage. All experiments were conducted at room temperature (25 ± 2 °C).

The residual concentrations of Mn^2+^, Fe^3+^, and Pb^2+^ ions were determined using an Agilent 7700 ICP-MS. Calibration standards with known concentrations of each metal ion were prepared, and appropriate internal standards were employed to correct for instrumental drift and matrix effects. Samples and calibration standards were introduced into the ICP-MS system for trace-level analysis, where the elements were ionized in the plasma and detected according to their characteristic mass-to-charge ratios (m/z). Quantification of the metal ions was performed using external calibration curves^31^.

#### Effect of pH on the adsorption process

To investigate the effect of pH on the adsorption process, 25.0 mg of the nanosorbents (HAp, CT, and HAp@CT) were separately added to 100.0 mL of aqueous solutions containing 12.0 ppm of Mn^2+^, Fe^3+^, or Pb^2+^ ions. The pH of the solutions was adjusted within the range of 2.0–10.0 using dilute HCl or NaOH solutions. The suspensions were shaken for 60.0 min at 150.0 rpm using a mechanical shaker. After equilibrium, the solid phase was separated by filtration using Whatman filter paper, and the residual concentrations of Mn^2+^, Fe^3+^, and Pb^2+^ ions were determined using an Agilent 7700 ICP-MS. The adsorption capacity, q (mg g^−1^), representing the amount of metal ions adsorbed onto the surface of the nanosorbents, was calculated using the following equation:

1 $$q{\text{ }}={\text{ }}({C_{\mathrm{o}}} - {C_{\mathrm{e}}}){\text{ }}V/m$$

where C_o_ and C_e_ (mg L^−1^) are the initial and equilibrium concentrations of the metal ions, V (L) is the volume of the solution, and m (g) is the mass of the adsorbent.

#### Effect of contact time on the adsorption process

The effect of contact time on the adsorption of Mn^2+^, Fe^3+^, and Pb^2+^ ions was investigated using 25.0 mg of the nanosorbents (HAp, CT, and HAp@CT), which were separately added to 100.0 mL of aqueous solutions containing 12.0 ppm of each metal ion. The pH of the solutions was adjusted to the optimal value determined in the previous pH optimization experiment. The mixtures were shaken at 150.0 rpm at room temperature for different contact times of 5.0, 10.0, 20.0, 30.0, 90.0, and 120.0 min using a mechanical shaker. After each predetermined contact time, the suspensions were filtered to separate the adsorbent, and the residual concentrations of Mn^2+^, Fe^3+^, and Pb^2+^ ions were determined using ICP-MS. The adsorption capacity for each condition was calculated using Eq. (1).

#### Effect of adsorbent dose on the adsorption process

The influence of adsorbent dosage on the removal efficiency of Mn^2+^, Fe^3+^, and Pb^2+^ ions was investigated using different masses of the nanosorbents (HAp, CT, and HAp@CT) ranging from 5.0 to 50.0 ± 1 mg. Each adsorbent dose was added separately to 100.0 mL of aqueous solutions containing 12.0 ppm of Mn^2+^, Fe^3+^, or Pb^2+^ ions. The experiments were conducted under the previously optimized pH and contact time conditions. The mixtures were shaken at 150.0 rpm at room temperature using a mechanical shaker. After equilibrium was reached, the suspensions were filtered, and the adsorption capacity was calculated using Eq. (1).

## Results and discussion

### Characterization of nano-sorbent and its pristine materials

#### FT-IR spectroscopy analysis before adsorption

**Fig. 1:**
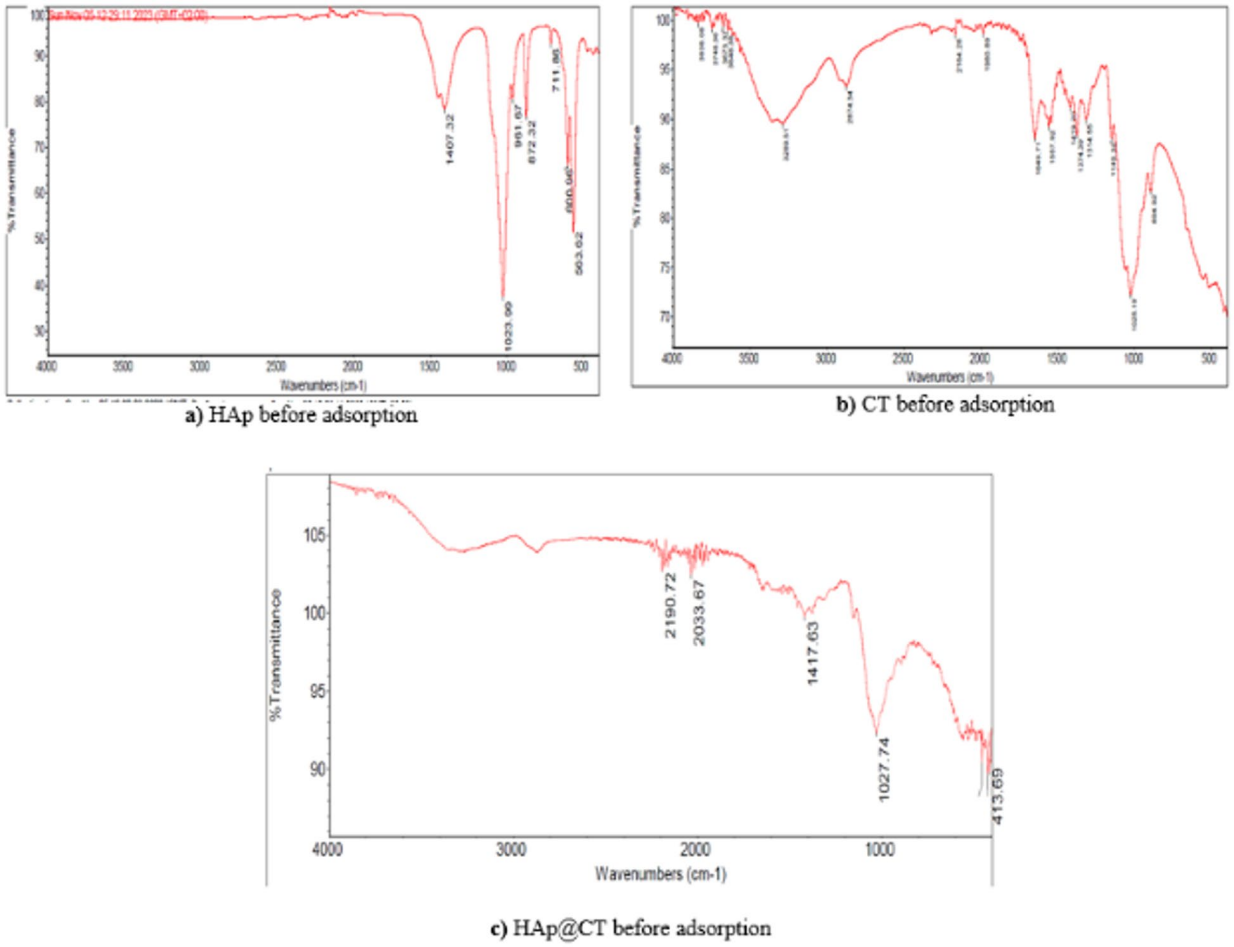
FT-IR spectra of the prepared adsorbents before adsorption: (**a**) HAp, (**b**) CT, and (**c**) HAp@CT composite.

The synthesized nanocomposite HAp@CT and its pristine components, HAp and CT, were characterized using FT-IR spectroscopy in the spectral range of 400–4000 cm^−1^. As shown in Fig. 1, the spectra exhibit several characteristic absorption bands corresponding to the functional groups present in the individual materials and their composite.

The FT-IR spectrum of HAp displays characteristic bands confirming its structural composition. A strong band at 1023.99 cm^−1^ corresponds to the asymmetric stretching vibration of phosphate groups (PO_4_^3−^), while the sharp band at 961.67 cm^−1^ is attributed to the symmetric stretching mode, both typical features of HAp. The presence of bands at 1407.32, 872.32, and 711.86 cm^−1^ indicates carbonate (CO_3_^2−^) substitution within the HAp lattice, commonly referred to as carbonated HAp. Additionally, the peaks at 563.62 and 600.96 cm^−1^ correspond to phosphate bending vibrations, which are typical of well-crystallized HAp structures. The incorporation of carbonate ions may influence the solubility and physicochemical properties of the material^31,32^.

The FT-IR spectrum of CT reveals several functional groups characteristic of CT. A broad and intense band at 3286.55 cm^−1^ corresponds to the overlapping stretching vibrations of O–H and N–H groups, indicating the presence of hydroxyl and amine functionalities. The peak at 2872.90 cm^−1^ is attributed to C–H stretching vibrations associated with the aliphatic chains of glucosamine units. The absorption band at 1645.09 cm^−1^ corresponds to the amide (I) band arising from C=O stretching of residual acetyl groups, while the peak at 1557.32 cm^−1^ represents the amide (II) band related to N–H bending and C–N stretching vibrations. These bands confirm the presence of primary amine groups generated during the deacetylation of chitin. Additional peaks at 1374.40 and 1312.31 cm^−1^ are attributed to C–H bending and C–N stretching vibrations, respectively. The bands observed at 1150.91 and 1025.81 cm^−1^ correspond to C–O–C and C–O stretching vibrations, characteristic of glycosidic bonds within the polysaccharide backbone. Moreover, the peak at 895.70 cm^−1^ indicates β-glycosidic linkages, confirming the polymeric structure of CT. Overall, these spectral features verify the successful formation of CT and the presence of its characteristic functional groups^33,34^.

Finally, the FT-IR spectrum of the HAp@CT nanocomposite exhibits absorption bands corresponding to both HAp, and CT, confirming the successful formation of the composite material. The band at 1027.74 cm^−1^ corresponds to the asymmetric stretching vibration of (PO_4_^3−^), characteristic of HAp, while the band at 413.69 cm^−1^ is attributed to phosphate bending vibrations. The absorption band at 1417.63 cm^−1^ may be associated with carbonate substitution within the HAp lattice or with C–H bending vibrations originating from CT. The broad band at 3286.55 cm^−1^ can be assigned to hydrogen bonding between the –OH groups of HAp and the –OH/–NH groups of CT. The absence or weakening of the bands at 1645.09 and 1557.32 cm^−1^ may be attributed to the relatively low CT content or to strong interactions between HAp, and CT, which may cause band shifting or broadening. Overall, the FT-IR spectrum confirms the successful formation of the HAp@CT nanocomposite through the presence of characteristic bands from both pristine materials^31–34^.

#### FT-IR analysis after adsorption

**Fig. 2:**
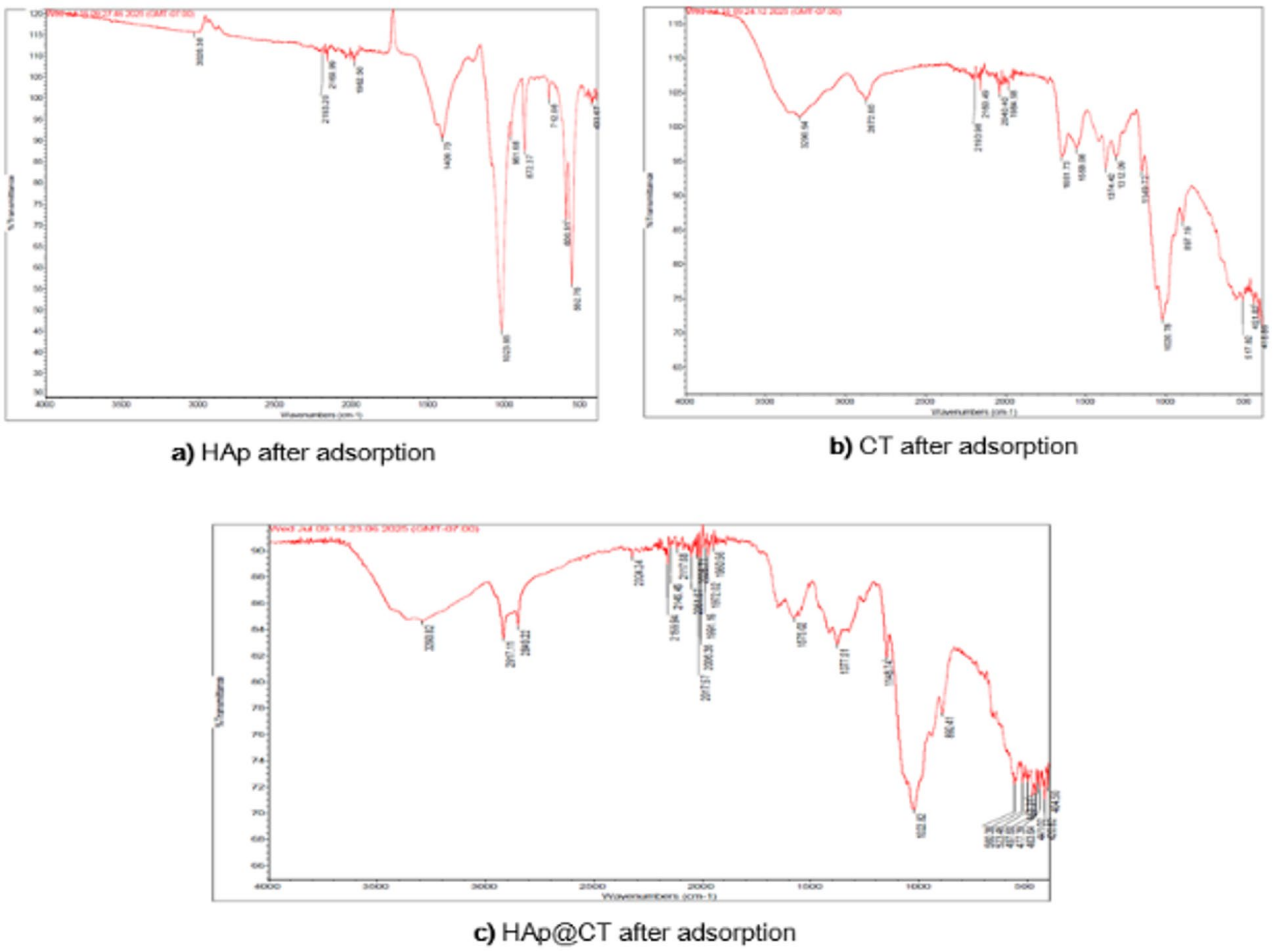
FT-IR spectra of the prepared adsorbents after adsorption: (**a**) HAp, (**b**) CT, and (**c**) HAp@CT composite.

The FT-IR spectra recorded after the adsorption of Mn^2+^, Fe^3+^, and Pb^2+^ ions as shown in Fig. 2, the spectra exhibit noticeable changes in several characteristic bands of HAp, CT, and the HAp@CT nanocomposite. In hydroxyapatite, slight shifts of (PO_4_^3−^) vibrational bands toward lower wavenumbers were observed after adsorption. These changes may indicate the involvement of phosphate groups in metal binding through surface complexation or partial ion-exchange processes, as commonly reported for calcium phosphate materials^35^. In addition, minor variations in the hydroxyl-related bands suggest possible surface hydration effects and the formation of hydrated metal species during adsorption^36^. These observations indicate that phosphate sites play an important role in metal uptake within HAp-based systems.

For CT, modifications in the amine (–NH_2_), and hydroxyl (–OH) related bands were observed after adsorption. These spectral changes may result from coordination interactions between metal ions and electron-donating functional groups present in the polymer matrix^37,38^. Similar behavior has been widely reported for CT-based adsorbents, where CT mechanisms dominate heavy-metal removal processes^36,39^.

In the HAp@CT nanocomposite, simultaneous changes in both phosphate- and amine-related bands were detected, suggesting that adsorption involves contributions from both the inorganic and polymeric components. This cooperative interaction mechanism has been previously proposed for HAp-CT composites used in heavy-metal remediation^35,40^.

Overall, the FT-IR results suggest that metal adsorption onto the composite occurs through a combination of phosphate coordination, ion exchange, and amine-mediated complexation, consistent with mechanisms reported in recent studies^38–40^.

#### X-ray diffraction analysis

**Fig. 3:**
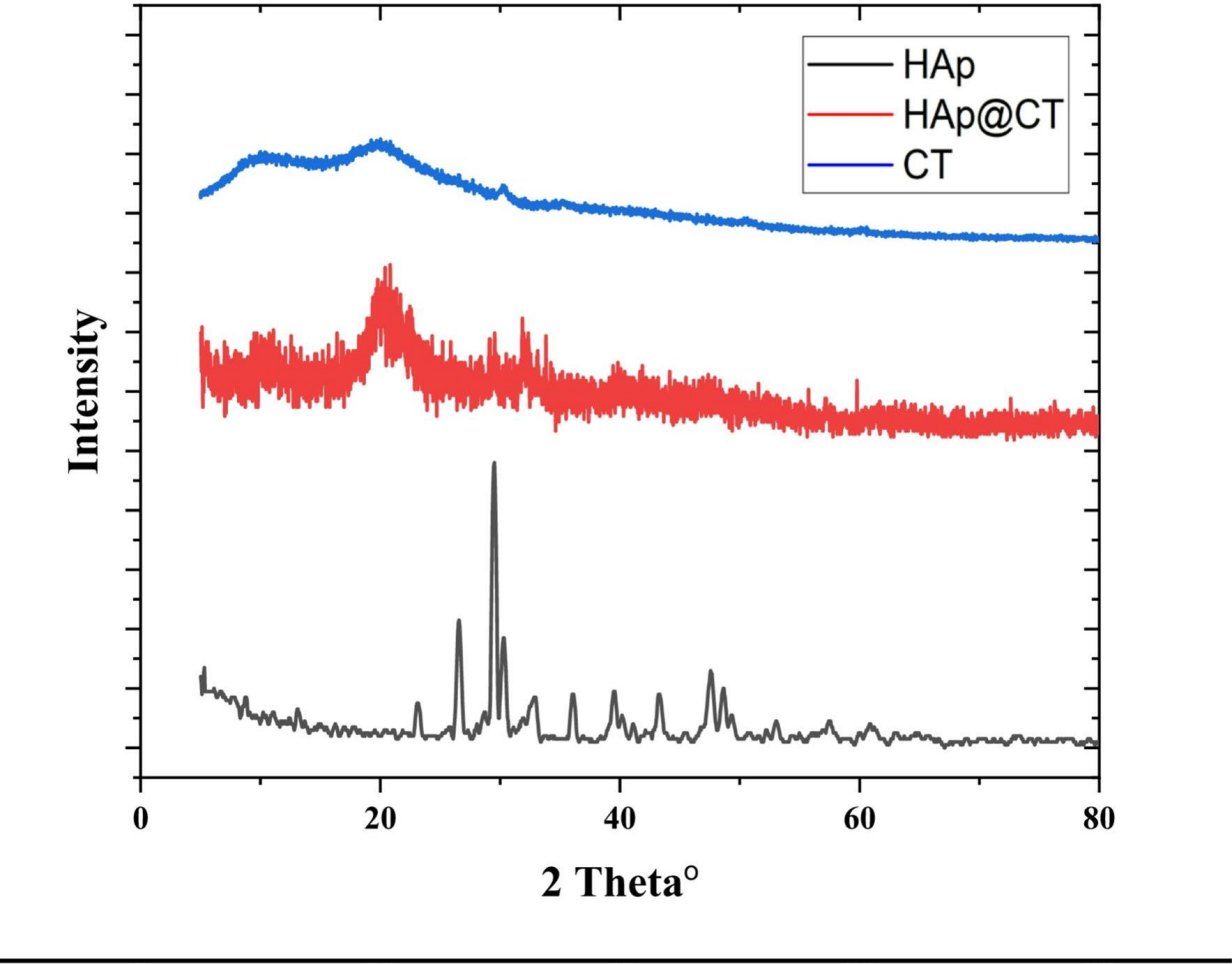
XRD patterns of HAp, CT, and their nanocomposite HAp@CT.

XRD analysis was performed to investigate the crystalline structure of HAp, CT, and the synthesized HAp@CT nanocomposite to confirm the successful formation of the composite material. The obtained diffraction patterns verified the presence of the individual components as well as their composite structure. As shown in Fig. 3, the XRD pattern of the HAp sample exhibits a well-defined crystalline structure, evidenced by several sharp and intense diffraction peaks across the 2θ range. The most prominent peaks appear at approximately 2θ = 25.9°, 31.7°, 32.9°, 34.0°, 39.8°, 46.7°, 49.5°, and 53.1°, which correspond to the characteristic diffraction planes of HAp. These reflections can be indexed to the crystallographic planes (002), (211), (112), (300), (310), (222), (213), and (004), respectively, which are consistent with the standard JCPDS card for pure HAp (JCPDS No. 09-0432). The high intensity and sharpness of these peaks indicate a high degree of crystallinity. Furthermore, the absence of additional diffraction peaks suggests phase purity, with no detectable secondary phases such as tricalcium phosphate or calcium oxide. Overall, the XRD pattern confirms the formation of a single-phase crystalline HAp structure, similar to naturally occurring apatite found in bone^31,32^.

The XRD pattern of pure CT exhibits a semicrystalline structure characterized by two broad diffraction peaks located at approximately 2θ ≈ 10° and 20°. The peak near 10° is associated with the (020) plane, representing hydrated crystalline regions of CT, while the stronger peak around 20° corresponds to the (110) plane, which arises from the regular arrangement of polymer chains stabilized through intra-intermolecular hydrogen bonding. These peaks reflect the partially ordered nature of CT, which contains both crystalline and amorphous domains. The intensity and sharpness of these peaks may vary depending on factors such as the degree of deacetylation, molecular weight, preparation method, and drying conditions^32–34^.

The XRD pattern of the HAp@CT nanocomposite displays broad bands within the 2θ range of approximately 10–30°, which is characteristic of the semi-crystalline or amorphous structure of CT. This broad feature overlaps with the low-angle diffraction peaks of HAp, particularly the (002) reflection near 25.9°, making individual peaks less distinguishable. Compared with pure HAp, the composite exhibits a noticeable reduction in peak intensity and sharpness, indicating a decrease in overall crystallinity. This reduction can be attributed to the nanoscale dispersion of HAp particles within the CT matrix and the strong interfacial interactions between the two components, which may hinder crystal growth. Nevertheless, a weak peak around 31.7° corresponding to the (211) plane of HAp can still be observed, confirming the presence of the apatite phase within the composite. Importantly, no additional peaks related to secondary phases such as tricalcium phosphate or calcium oxide were detected, indicating the phase purity of the prepared composite. These results confirm the successful formation of the HAp@CT nanocomposite with HAp particles uniformly distributed within the CT matrix^31–34^.

#### Thermal gravimetric analysis (TGA)

**Fig. 4:**
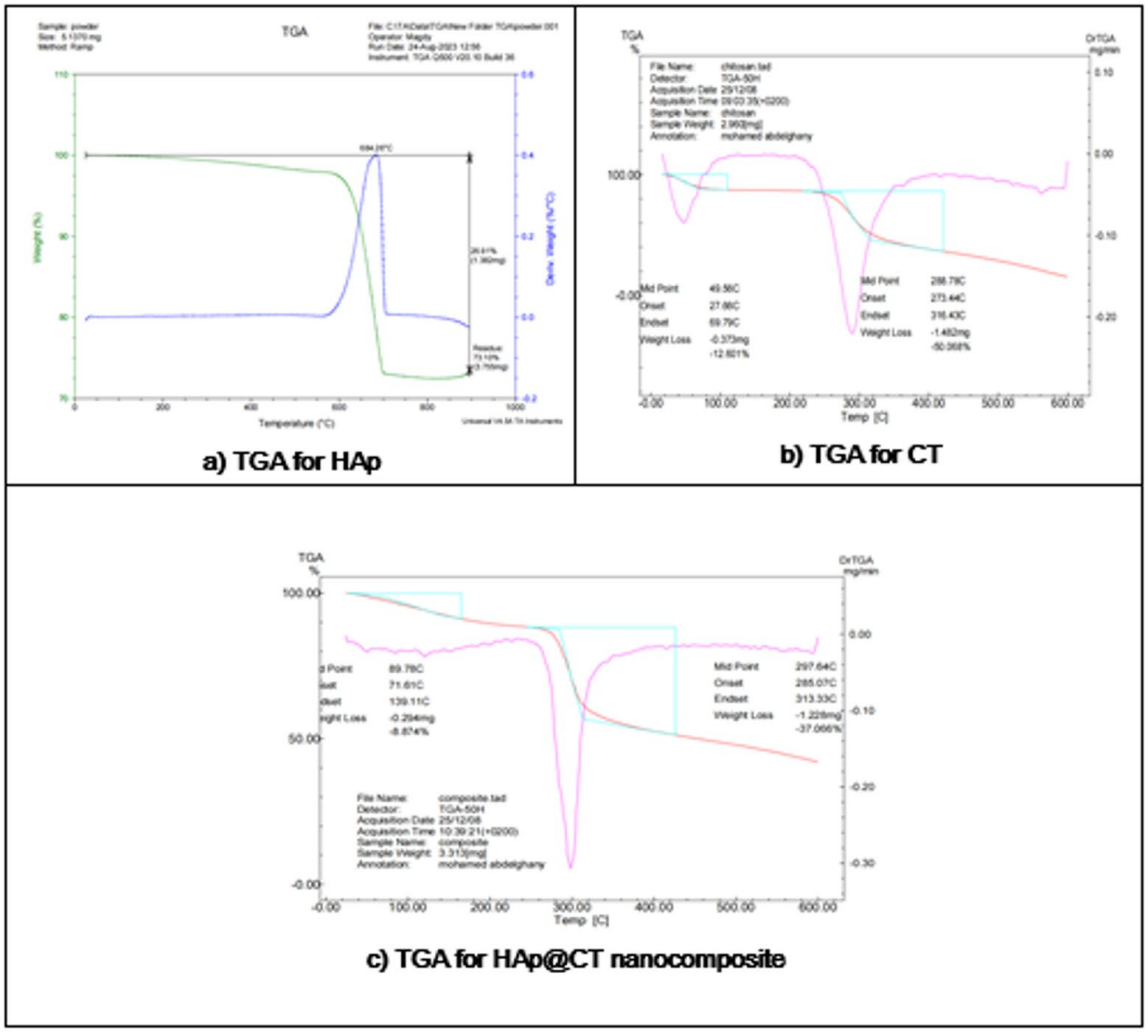
(**a–c**) TGA for HAp, CT, and their nanocomposite HAp@CT.

Thermogravimetric analysis (TGA) was conducted for HAp, CT, and the HAp@CT nanocomposite to investigate their thermal degradation behavior and evaluate their thermal stability over a wide temperature range, as illustrated in Fig. 4a–c. The TGA profile of HAp demonstrates its high thermal stability. Only minor changes in the weight% were observed with increasing temperature, indicating that HAp does not undergo significant decomposition or phase transformation up to approximately 684.26 °C. The total weight loss recorded over the entire temperature range was about 26.91%, which may be attributed mainly to the removal of adsorbed moisture and structural water. This behavior reflects the inherent stability of the crystalline calcium phosphate framework of HAp. Such thermal stability makes HAp a promising candidate for applications that require high thermal resistance, including ceramic membranes and other materials used in industrial wastewater treatment processes.

The TGA curve of CT exhibits a multi-step thermal degradation pattern typical of CT-based polymers. The first weight-loss stage, approximately 12.60%, occurs between 27.66 °C and 69.79 °C, with a maximum centered around 49.56 °C. This stage is attributed to the evaporation of physically adsorbed and bound water molecules due to the hygroscopic nature of CT. The major degradation stage occurs in the temperature range of 273.44–316.43 °C and corresponds to the thermal decomposition of the CT polymer backbone. This process involves depolymerization reactions, cleavage of glycosidic linkages, and decomposition of functional groups within the polysaccharide structure. Such degradation behavior is commonly observed for CT materials, where the primary thermal decomposition typically occurs above 200 °C.

The TGA curve of the HAp@CT nanocomposite exhibits multiple degradation stages resulting from the combined contributions of both HAp, and CT. The first weight-loss stage occurs between 71.6 °C and 139.11 °C with a mass loss of approximately 8.87%, which is attributed to the removal of physically adsorbed water associated with the hydrophilic –OH and –NH_2_ groups present in CT. The second and main degradation step appears in the temperature range of approximately 285.07–313.33 °C, accompanied by a significant weight loss of about 37.06%. This stage corresponds to the thermal decomposition of the CT backbone through depolymerization, cleavage of glycosidic bonds, and degradation of amino and acetyl functional groups. Notably, the relatively higher temperature of this degradation step compared to pure CT suggests enhanced thermal stability due to the interactions between HAp, and CT, including hydrogen bonding and interfacial interactions within the composite structure. Above 330 °C, a gradual mass loss continues up to approximately 600 °C, which can be attributed to further decomposition and carbonization of residual CT components, while the remaining mass corresponds mainly to the thermally stable HAp phase. Overall, the TGA results confirm that the incorporation of HAp improves the thermal stability of CT by increasing the residual inorganic content and strengthening the structural integrity of the nanocomposite.

#### Transmission electron microscopy (TEM)

**Fig. 5:**
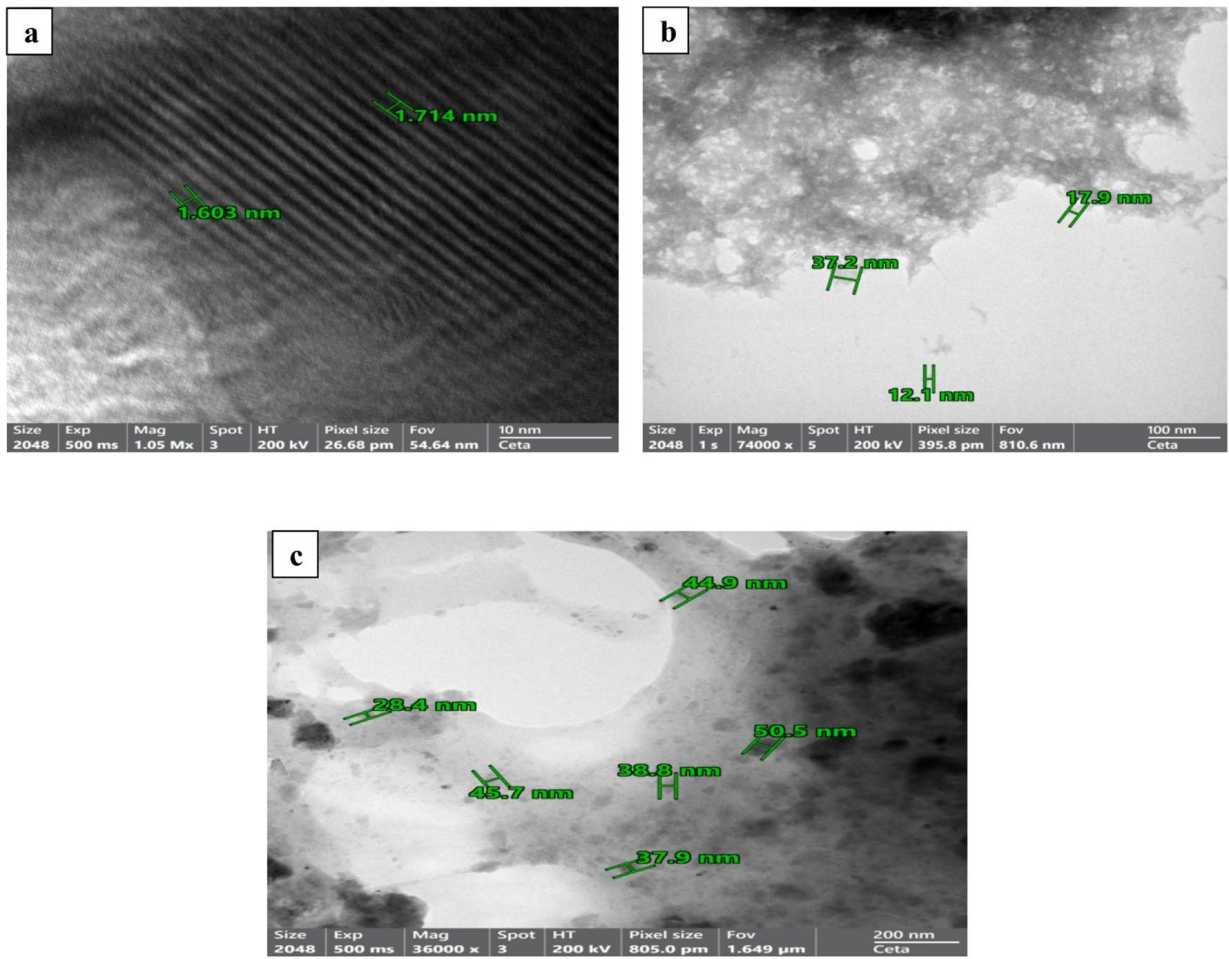
TEM images of (**a**) HAp, (**b**) CT, and (**c**) HAp@CT nanocomposite.

The surface morphology and nanostructure of the synthesized nanosorbent and its pristine materials were investigated using transmission electron microscopy (TEM), as illustrated in Fig. 5a–c. The TEM image of HAp presented in Fig. 5a reveals clear and well-defined lattice fringes, indicating the highly crystalline nature of the material. The observed interplanar spacings of approximately 1.603 nm and 1.714 nm correspond to specific crystallographic planes of HAp, confirming the presence of ordered atomic arrangements characteristic of the hexagonal crystal structure of HAp.

In contrast, the TEM image of CT, shown in Fig. 5b, displays an amorphous and irregular aggregated morphology. The structure consists of interconnected film-like regions and porous clusters typical of polymeric CT materials. Darker regions in the micrograph correspond to relatively dense areas, whereas lighter regions represent thinner or less compact domains. Such porous and irregular morphology suggests a relatively high surface area, which can enhance the adsorption performance of CT-based materials. The TEM analysis also confirms the nanoscale dimensions of the CT structure, with particle sizes of approximately 5.31 nm.

The TEM image of the HAp@CT nanocomposite Fig. 5c illustrates the successful integration of HAp within the CT matrix. The micrograph reveals a heterogeneous and porous structure in which CT appears as a light, diffuse, fibrous matrix, while the darker and denser regions correspond to HAp nanoparticles. These HAp particles are well distributed throughout the CT network, indicating effective incorporation and surface anchoring of HAp within the polymer matrix. The porous and loosely packed structure of the composite, inherited from the CT framework, may facilitate improved dispersion of HAp particles and provide additional active sites, thereby potentially enhancing the adsorption performance of the nanocomposite.

#### Scanning electron microscopy (SEM) after the adsorption process

**Fig. 6:**
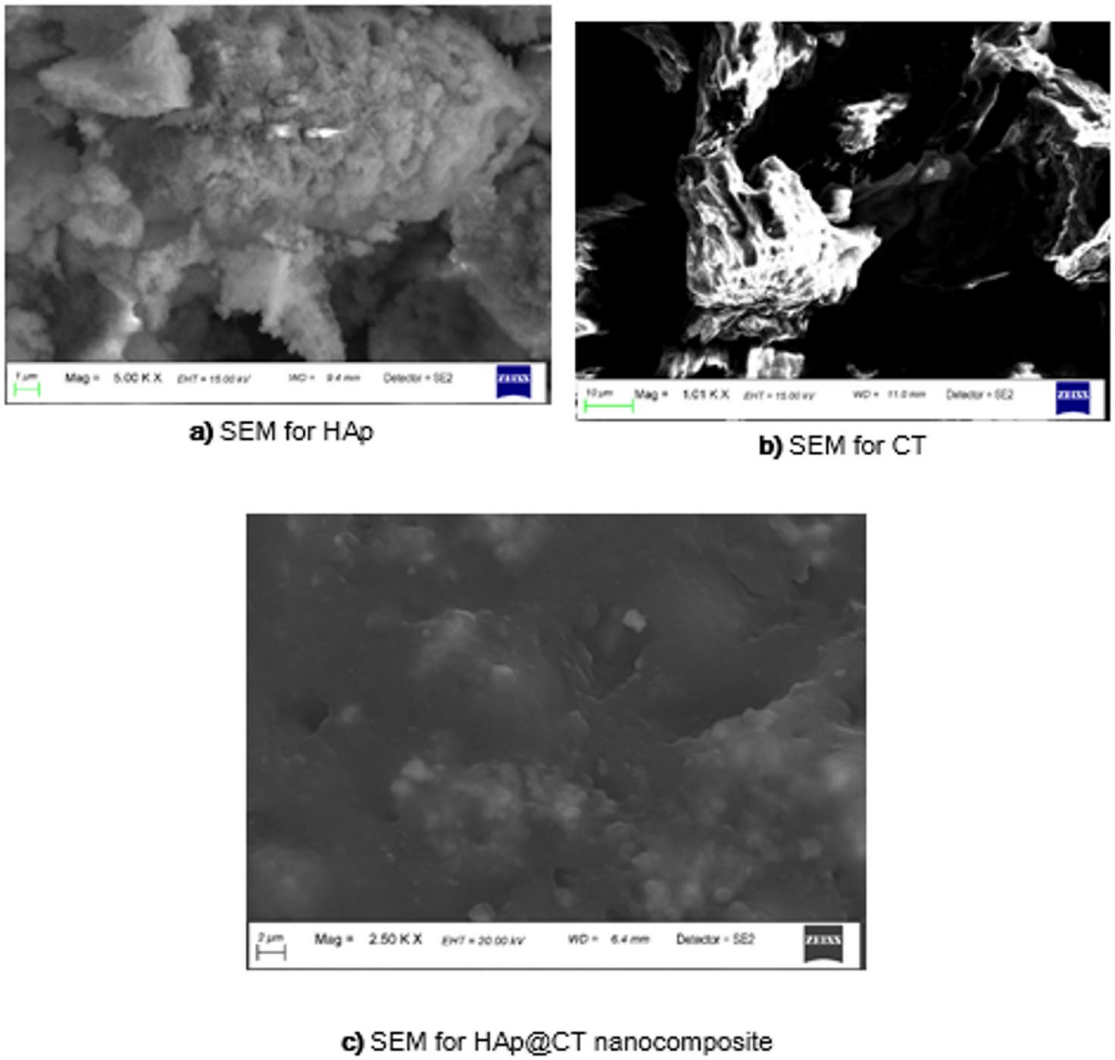
SEM images after the adsorption for (**a**) HAp, (**b**) CT, and (**c**) HAp@CT nanocomposite.

The surface morphology of the nano-adsorbents after the adsorption of Mn^2+^, Fe^3+^, and Pb^2+^ ions was investigated using SEM, as presented in Fig. 6a–c. The HAp adsorbent exhibits a dense and agglomerated crystalline structure. After adsorption, the surface appears rougher with visible particulate deposits, which may indicate successful metal ion attachment and possible surface precipitation, as commonly reported for HAp -based adsorbents during heavy-metal uptake^41,42^.

In contrast, the CT adsorbent displays a fibrous and highly porous network structure. After exposure to the metal ions, this porous morphology becomes partially occluded, with noticeable clusters and a smoother texture along the polymer strands. These morphological changes suggest that metal ions interact with the polymer matrix and may occupy the available pores within the CT structure, consistent with previous studies on CT -based adsorption systems^39,40,42^.

The HAp@CT nanocomposite exhibits a distinct synergistic morphology, where HAp particles are embedded and well dispersed within the fibrous CT matrix. This hybrid structure provides a relatively high surface area and multiple active sites for adsorption. After adsorption, the composite surface shows relatively uniform coverage of deposited material without significant pore blockage, indicating efficient uptake of Mn^2+^, Fe^3+^, and Pb^2+^ ions. Similar morphological behavior has been reported for HAp@CT composite adsorbents used in heavy-metal removal^39–42^. Overall, the SEM observations confirm that the nanocomposite successfully integrates the structural characteristics of both components, maintaining morphological stability while facilitating enhanced metal ion adsorption compared with the individual HAp and CT materials^39–42^.

#### Energy-dispersive X-ray (EDX)

**Fig. 7:**
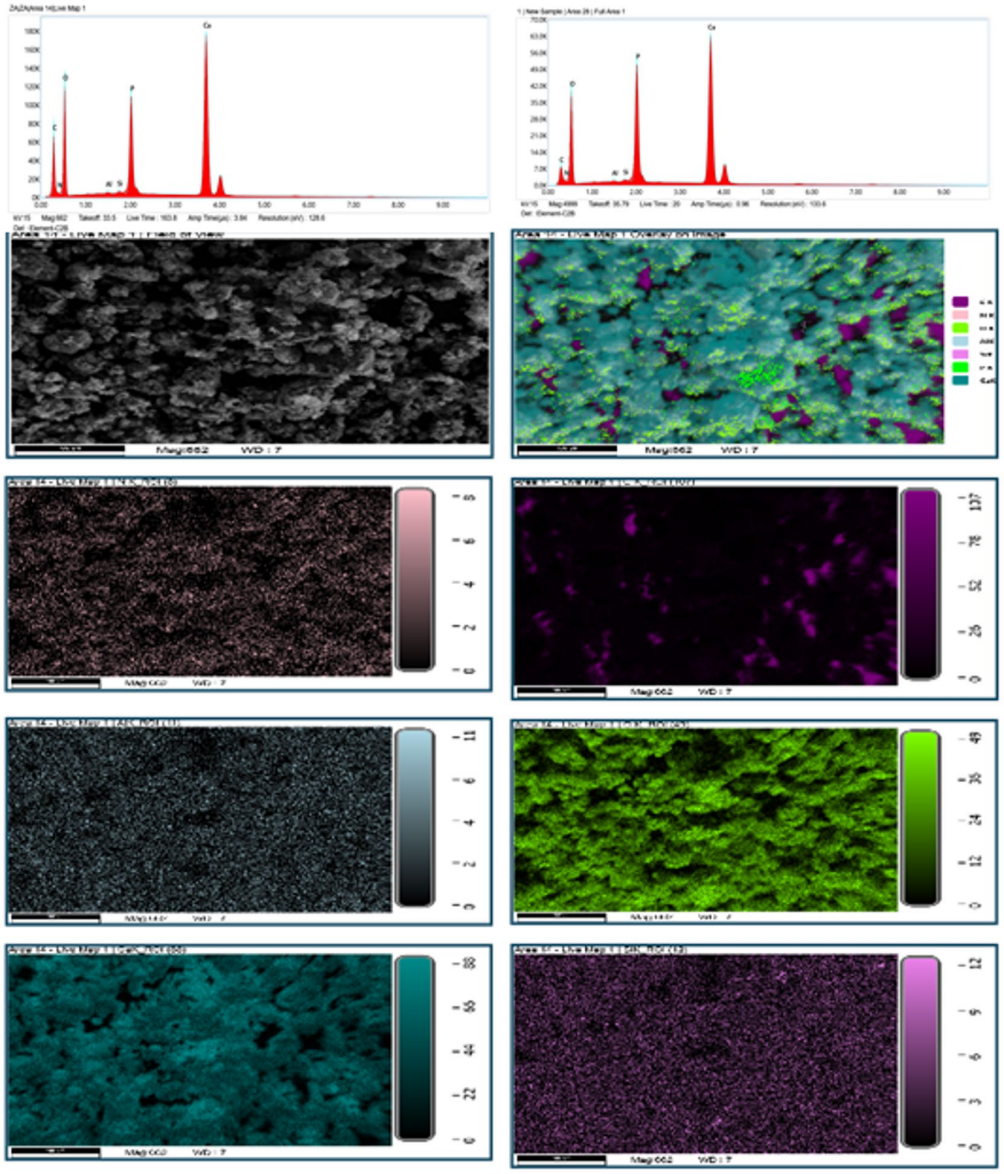

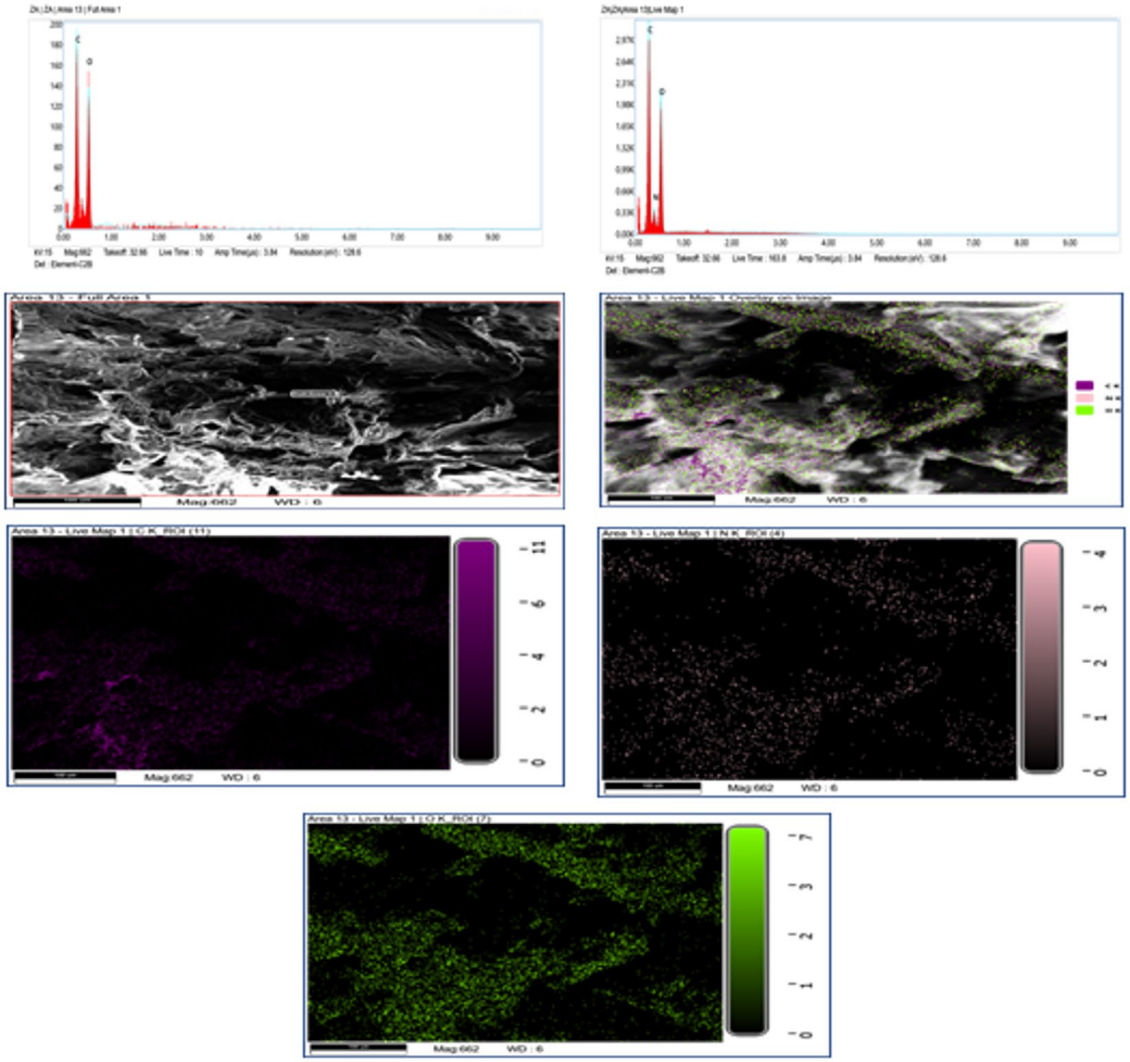

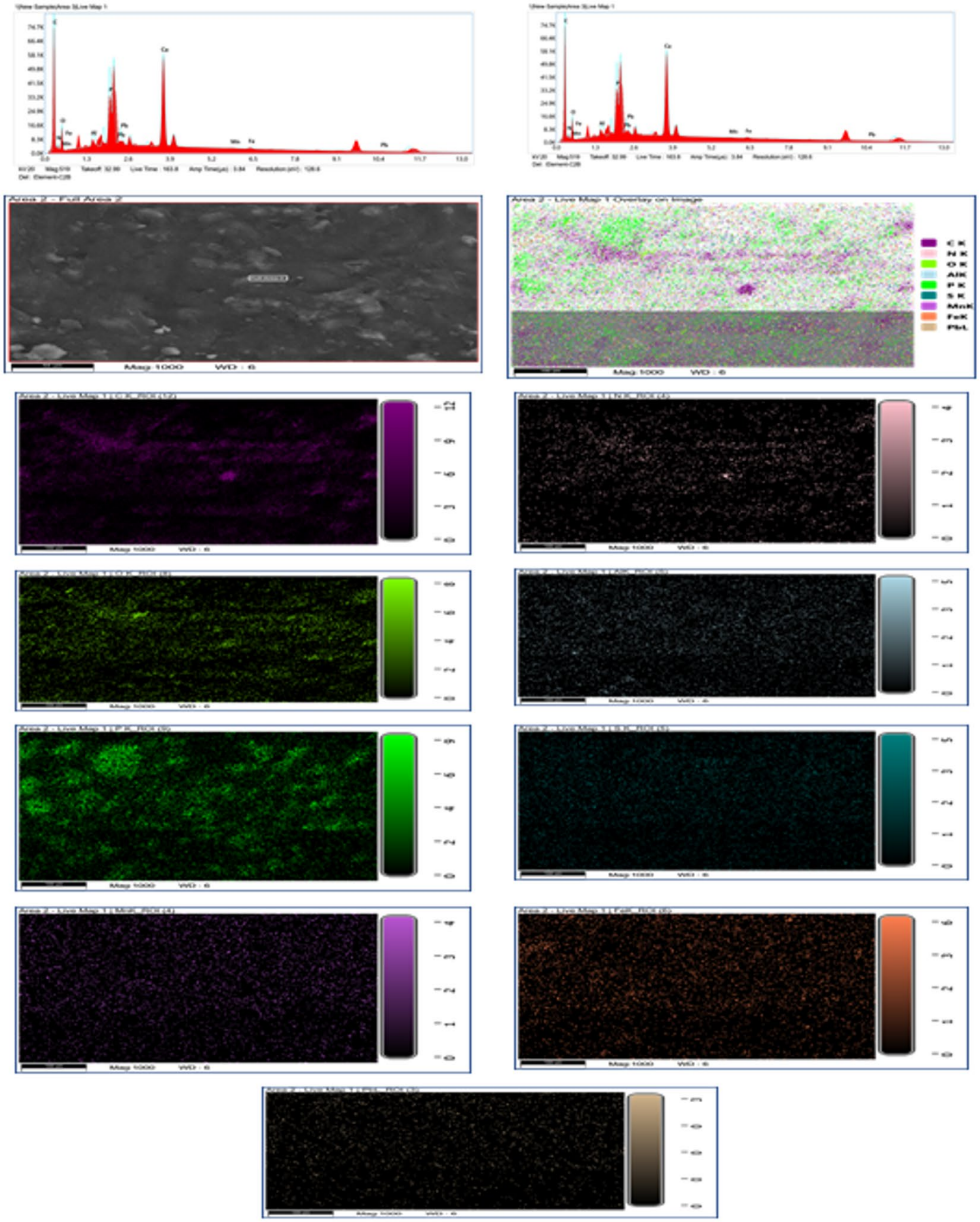
(**a**) EDX, and SEM images of HAp after the adsorption. (**b**) EDX, and SEM images of CT after the adsorption. (**c**) EDX, and SEM images of for HAp@CT nanocomposite after the adsorption.

Energy-dispersive X-ray (EDX) analysis was performed to investigate the surface elemental composition of HAp, CT, and HAp@CT after the adsorption process and to confirm surface modification resulting from the interaction with the adsorbed species, as illustrated in Fig. 7a–c^43,44^. The EDX spectra and corresponding SEM images of HAp after adsorption indicate that carbon and oxygen were the predominant elements on the HAp surface. Carbon was detected at 48.7 wt% and oxygen at 51.3 wt% in one analyzed region, indicating the dominance of oxygen-containing functional groups such as phosphate and hydroxyl groups, which play a significant role in metal binding^43,44^. In another surface region, carbon, oxygen, and nitrogen were identified with weight percentages of 44.3 wt%, 44.6 wt%, and 11.1 wt%, respectively. The appearance of nitrogen after adsorption may indicate the attachment of nitrogen-containing species, which could be associated with metal–ligand complexes or counter-ions accompanying heavy-metal adsorption^44,46^. The consistently high oxygen content observed in both spectra suggests the participation of hydroxyl and phosphate groups in the adsorption process. These findings indicate that heavy-metal uptake on HAp may occur primarily through surface complexation and chemical interactions rather than purely physical adsorption^43,44^. The slight variation in elemental composition between the analyzed regions also reflects the surface heterogeneity of the adsorbent after adsorption.

The EDX analysis of CT after the adsorption of Mn^2+^, Fe^3+^, and Pb^2+^ ions confirm the successful uptake of these metal ions onto the CT surface. The spectra are dominated by carbon, oxygen, and nitrogen, which are characteristic elements of CT^43,44^. Carbon was detected in the range of 54.2–64.8 wt%, oxygen at 15.3–17.0 wt%, and nitrogen at 6.0–7.1 wt%, confirming the structural integrity of the CT backbone after adsorption. The presence of nitrogen is particularly significant because it originates from amine (–NH_2_) groups that serve as important active sites for metal binding through coordination and electrostatic interactions^43,44^. The EDX spectra also reveal the presence of the target heavy metals, with Pb detected up to 2.7 wt%, Fe up to 0.5 wt%, and Mn up to 0.5 wt%, providing direct evidence of successful adsorption of Mn^2+^, Fe^3+^, and Pb^2+^ ions onto the CT surface. Minor amounts of phosphorus and calcium may be attributed to residual interactions or traces from composite preparation or adsorption media, while elements such as Al, Si, Na, and K are likely associated with background signals or experimental conditions. Overall, the EDX results indicate that CT effectively adsorbs Mn^2+^, Fe^3+^, and Pb^2+^ ions primarily through its amine and hydroxyl functional groups, suggesting that the adsorption mechanism is dominated by chemical complexation rather than simple physical adsorption^43,44^.

The EDX analysis of the HAp@CT nanocomposite after the adsorption of Mn^2+^, Fe^3+^, and Pb^2+^ ions further confirm the successful uptake of metal ions onto the adsorbent surface. The spectra reveal the coexistence of elements originating from both HAp and CT, as well as the presence of newly adsorbed metal species. Calcium and phosphorus were detected at 31.4 wt% and 11.0 wt%, respectively, confirming the structural stability of the HAp phase after adsorption^43–46^. Carbon, oxygen, and nitrogen were also identified with weight percentages of 18.7 wt%, 36.9 wt%, and 1.4 wt%, respectively, which are characteristic of the CT matrix and particularly highlight the presence of amine (–NH_2_) and hydroxyl (–OH) functional groups^43–46^. These functional groups, together with phosphate groups from HAp, act as active binding sites for Mn^2+^, Fe^3+^, and Pb^2+^ ions through surface complexation, electrostatic attraction, and possible ion-exchange mechanisms with Ca^2+^^43–46^. The persistence of Ca and P peaks alongside organic elements indicates that metal adsorption occurs mainly through surface interactions without destruction of the composite framework. Overall, the EDX data confirm the successful adsorption of Mn^2+^, Fe^3+^, and Pb^2+^ ions onto the HAp@CT composite and demonstrate the synergistic role of CT functional groups and HAp phosphate sites in enhancing heavy-metal removal through chemical adsorption mechanisms^43–46^.

### Adsorption of Mn^2+^, Fe^3+^, and Pb^2+^ from water onto HAp, CT, and HAp@CT nanosorbents

#### Effect of pH on adsorption performance

**Fig. 8:**
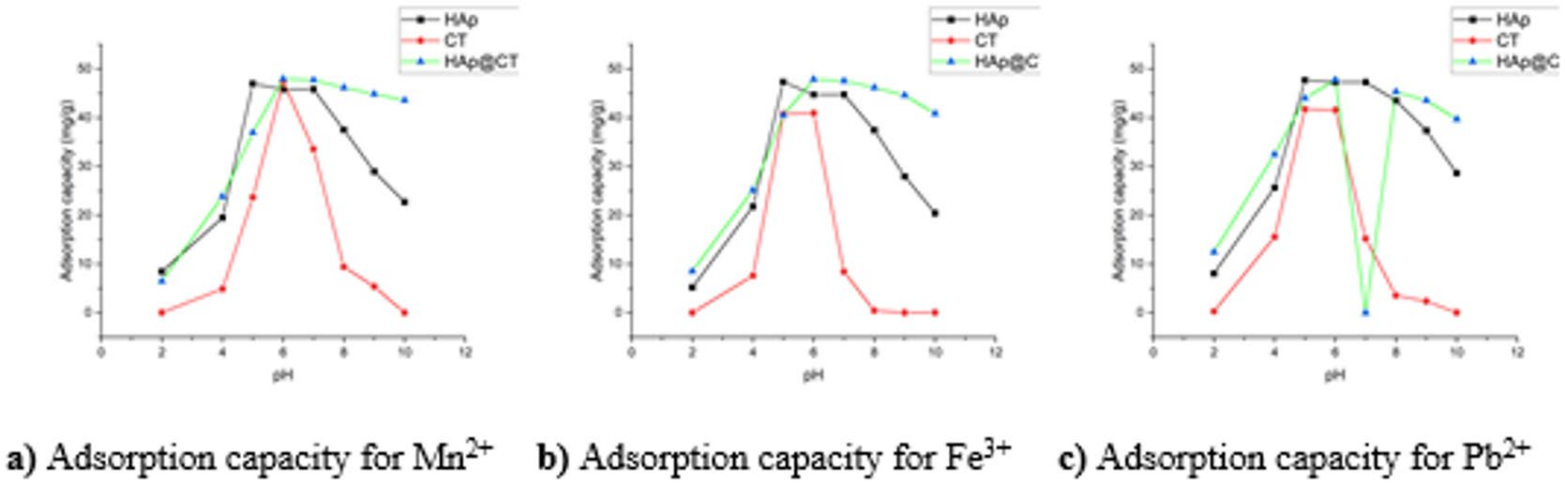
(**a–c**) Effect of solution pH on the adsorption capacity using HAp, CT, and HAp@CT Mn^2+^, Fe^3+^, and Pb^2+^ respectively.

The influence of solution pH on adsorption performance was investigated because pH is one of the most critical parameters controlling both the surface charge of the adsorbent and the speciation of metal ions in solution. Adsorption experiments were conducted over a pH range of 2.0–7.0 for the removal of Mn^2+^, Fe^3+^, and Pb^2+^ ions in order to determine the optimal pH conditions for adsorption under the applied experimental parameters. The adsorption behavior of Mn^2+^, Fe^3+^, and Pb^2+^ as a function of pH is presented in Fig. 8a–c. The improvement in adsorption efficiency with increasing pH can be attributed to the presence of several active functional groups on the nanosorbent surface. HAp provides (PO_4_^3−^) and hydroxyl (–OH) groups, while CT contributes amine (–NH_2_), hydroxyl (–OH), and acetamido (–NHCOCH₃) functional groups^42,45,46^. These groups facilitate metal-ion binding through electrostatic attraction and surface complexation mechanisms. As shown in Fig. 8a, the highest adsorption capacity for Mn^2+^ at the studied initial concentration was observed in the pH range of 5.0–6.0, reaching 46.96, 47.39, and 47.96 mg g^−1^ for HAp, CT, and HAp@CT, respectively. Similarly, Fe^3+^ adsorption Fig. 8b exhibited optimal performance within the same pH range, with adsorption capacities of 47.32, 40.92, and 47.84 mg g^−1^ for HAp, CT, and HAp@CT, respectively. In the case of Pb^2+^ Fig. 8c, the highest adsorption values were obtained at pH 6.0, reaching 47.29, 41.56, and 47.77 mg g^−1^ for HAp, CT, and HAp@CT, respectively.

The pH-dependent adsorption behavior can be explained by competitive interactions between hydrogen ions (H⁺) and metal cations for the available adsorption sites. At low pH values, the high concentration of H⁺ ions strongly compete with Mn^2+^, Fe^3+^, and Pb^2+^ ions for the active sites, resulting in reduced adsorption efficiency. As the pH increases, the concentration of H⁺ ions decrease, which weakens this competition and enhances the interaction between metal ions and the functional groups on the adsorbent surface^45,46^.

#### Zeta potential analysis

**Fig. 9:**
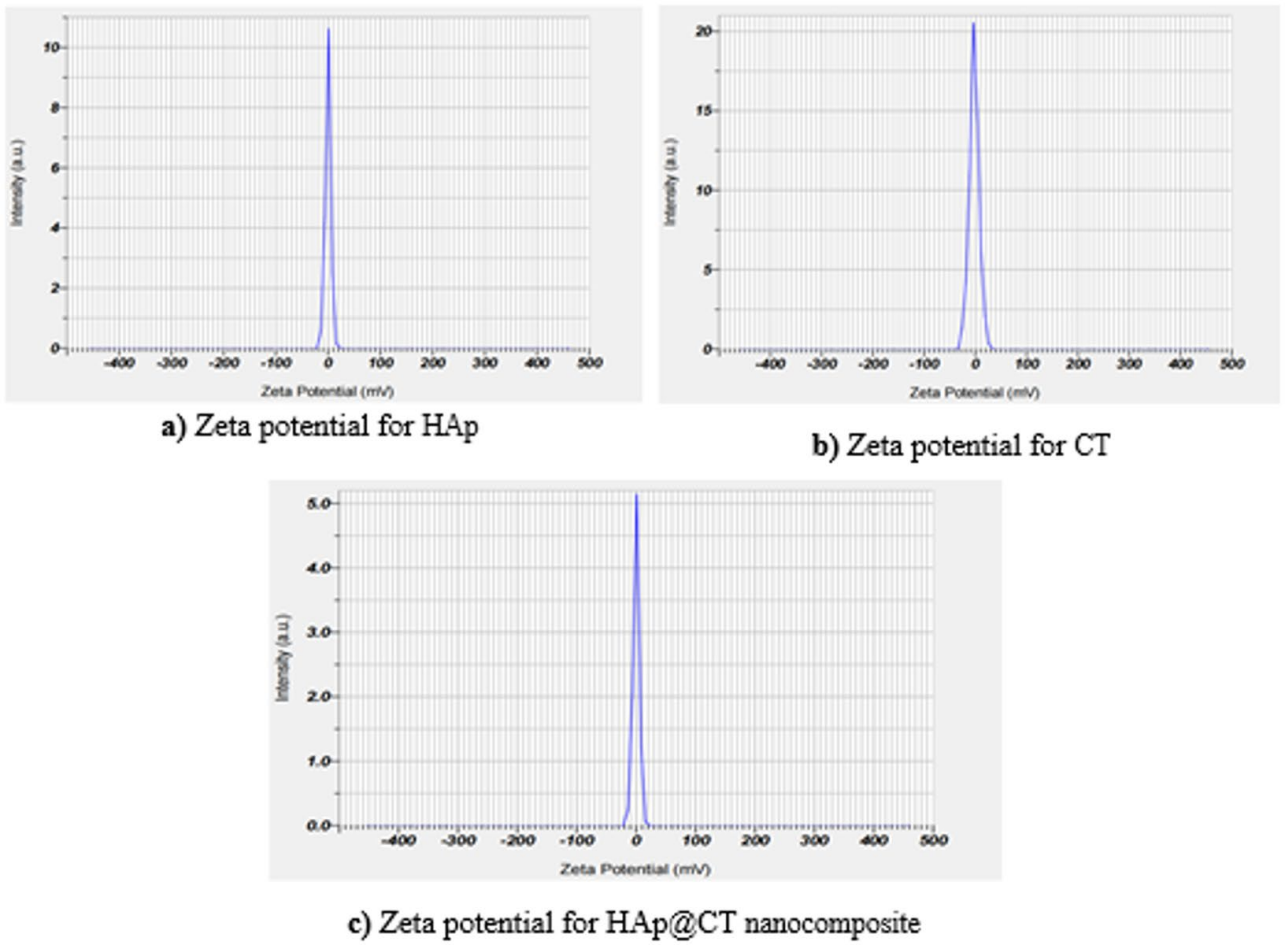
(**a–c**) Zeta Potential for HAp, CT, and HAp@CT nanocomposite.

Investigating the surface charge properties of the three adsorbents is essential for understanding their interaction mechanisms with heavy metal ions. The zeta potential measurements presented in Fig. 9a–c reveal distinct surface charge characteristics for each material^47,48^. HAp exhibited a positive surface charge within the investigated pH range, which is consistent with its surface chemistry under the applied experimental conditions^47^. In contrast, CT showed a negative surface potential at the tested pH values, indicating partial deprotonation of surface functional groups and the presence of negatively charged active sites^47,48^. The HAp@CT nanocomposite displayed an intermediate zeta potential value, reflecting the successful integration of both components and the modification of the overall surface chemistry of the composite material^47^. The altered surface charge of the nanocomposite enhances electrostatic interactions with metal ions and contributes to improved adsorption performance^47,48^. Although electrostatic attraction plays an important role in metal uptake, additional mechanisms such as surface complexation and ion exchange are also likely involved in the adsorption process^47,48^. The synergistic combination of HAp and CT provides a diverse distribution of functional groups, increasing the availability of active binding sites for metal ions. Consequently, the zeta potential results support the adsorption findings and confirm that the engineered surface properties of HAp@CT play a crucial role in its enhanced affinity toward Mn^2+^, Fe^3+^, and Pb^2+^ ions^47,48^.

#### Kinetic of the adsorption removal at different contact times

**Fig. 10:**
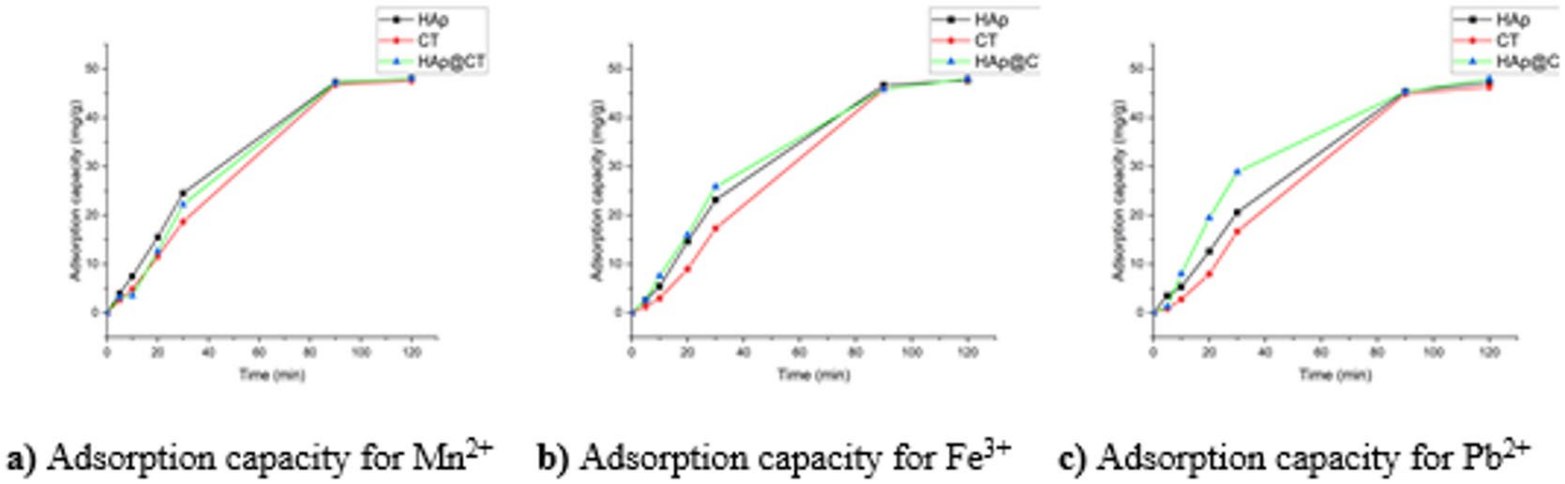
(**a–c**) Effect of contact time (min) on the adsorption capacity using HAp, CT, and HAp@CT for Mn^2+^, Fe^3+^, and Pb^2+^ respectively.

The adsorption of Mn^2+^, Fe^3+^, and Pb^2+^ ions were investigated over various contact times (5.0, 10.0, 12.0, 30.0, 90.0, and 120.0 min) using a batch method with an automatic shaker to ensure proper interaction between the metal ions in aqueous solution and the different nanosorbents, as shown in Fig. 10a–c. For Mn^2+^ ions, the adsorption capacity increased with contact time and reached to maximum values at 48.00, 47.57, and 48.05 mg g^−1^ after 120 min for HAp, CT, and HAp@CT, respectively. Similarly, Fe^3+^ adsorption increased gradually with time, reaching to 47.65, 47.98, and 48.00 mg g^−1^ at 120 min for HAp, CT, and HAp@CT, respectively. The same trend was observed for Pb^2+^ ions, where the adsorption capacities increased over time and reached 47.18, 46.27, and 47.95 mg g^−1^ for HAp, CT, and HAp@CT, respectively.

**Fig. 11:**
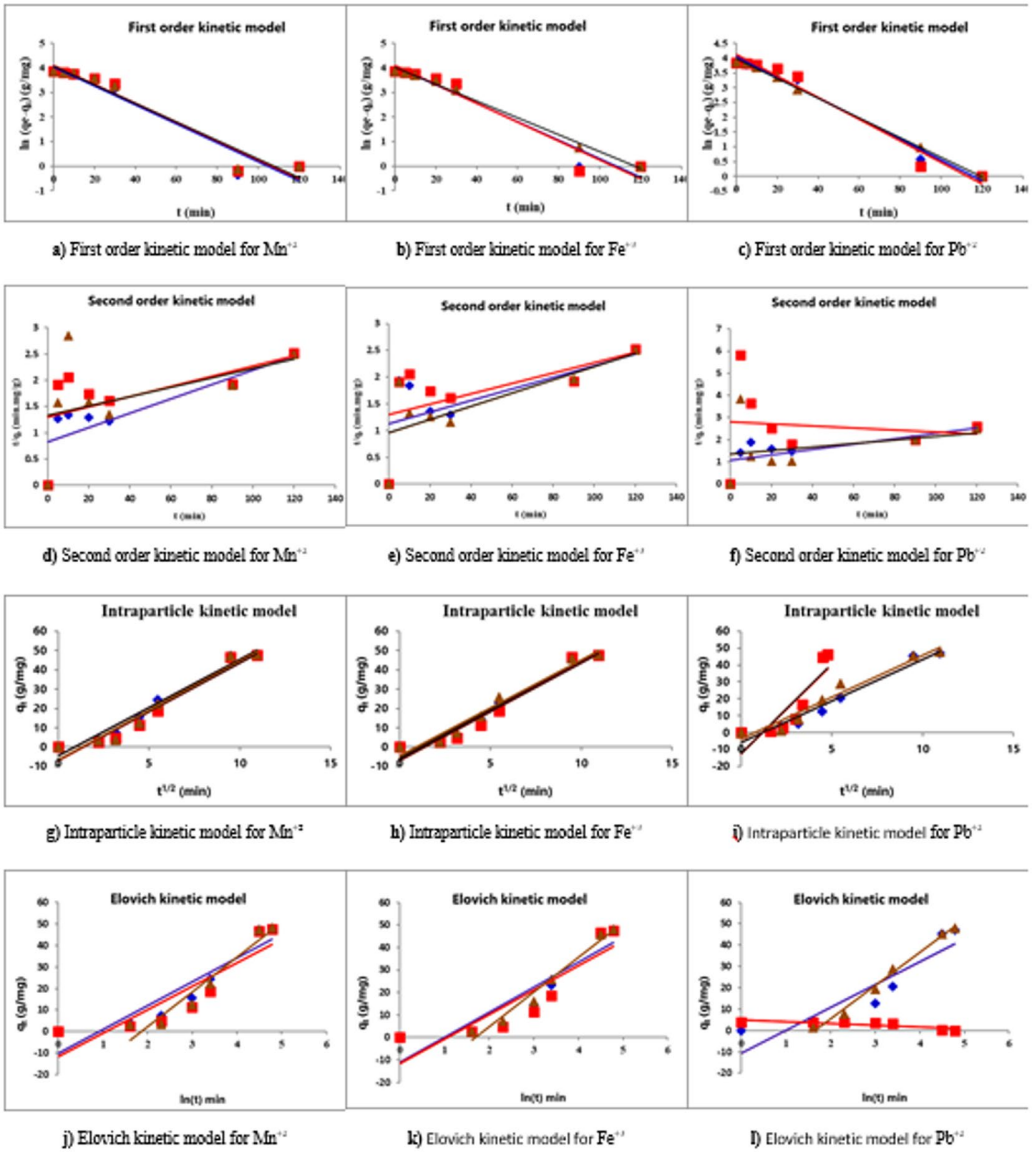
(**a–l**) Adsorption isotherm and kinetic models for HAp, CT, and HAp@CT nanocomposite for Mn^2+^, Fe^3+^, and Pb^2+^.

To investigate the adsorption mechanism, kinetic analysis was performed. The experimental data were fitted to four kinetic models: pseudo-first-order (PFO), pseudo-second-order (PSO), intraparticle diffusion, and Elovich models, as illustrated in Fig. 11a–l^49,50^. Initially, the pseudo-first-order (PFO) model was applied, which describes the relationship between time (t) and the adsorption process according to Eq. (2):

2 $${\text{ln }}\left( {{{\mathrm{q}}_{\mathrm{e}}} - {{\mathrm{q}}_{\mathrm{t}}}} \right)\,=\,{\text{ln }}{{\mathrm{q}}_{\mathrm{e}}}{\text{ }} - {\text{ }}{{\mathrm{k}}_1}{\mathrm{t}}$$

where q_t_ (mg g^−1^) represents the amount of Mn^2+^, Fe^3+^, or Pb^2+^ adsorbed at time t, q_e_ (mg g^−1^) is the adsorption capacity at equilibrium, and k₁ (min^−1^) is the rate constant of the PFO model.

The pseudo-second-order (PSO) model was also used to evaluate the adsorption kinetics and is represented by Eq. (3):

3 $${\mathrm{t}}/{{\mathrm{q}}_{\mathrm{t}}}=1/\left( {{{\mathrm{k}}_2}{{\mathrm{q}}_{\mathrm{e}}}^{2}} \right)\,+\,{\mathrm{t}}/{{\mathrm{q}}_{\mathrm{e}}}$$

where k_2_ (g mg^−1^ min^−1^) is the PSO rate constant and q_e_ is the equilibrium adsorption capacity. The intraparticle diffusion model was applied to determine whether the diffusion of metal ions within the pores of the adsorbent controls the adsorption process. In this model, adsorption occurs in two stages: the first stage involves the external diffusion of Mn^2+^, Fe^3+^, or Pb^2+^ ions toward the adsorbent surface, while the second stage involves diffusion into the internal pores of the nanosorbent. This model is expressed by Eq. (4):

4 $${{\mathrm{q}}_{\mathrm{t}}}{\text{ }}={\text{ }}{{\mathrm{k}}_{{\mathrm{id}}}}{{\mathrm{t}}^{1/2}}+C$$

where k_id_ (mg g^−1^ min^−1/2^) is the intraparticle diffusion rate constant and C represents the boundary layer thickness. The Elovich model, which assumes chemisorption on a heterogeneous surface, was also employed and is expressed in Eq. (5):

5 $${q_t}{\text{ }}={\text{ }}(1/\beta ){\text{ }}ln(\alpha \beta ){\text{ }}+{\text{ }}(1/\beta ){\text{ }}ln{\text{ }}t$$

where α (mg g^−1^ min^−1^) represents the initial adsorption rate and β (g mg^−1^) is related to the surface coverage and activation energy for chemisorption.

The adsorption kinetics were analyzed using the PFO, and PSO models as illustrated in Table 3. The PSO model provided a better fit (R^2^ > 0.98), indicating that chemisorption is likely the rate-limiting step. The relatively high value of the PSO rate constant (k_2_) suggests a strong affinity between Mn^2+^, Fe^3+^, and Pb^2+^ ions and the active sites of the composite. This behavior is consistent with electron-sharing or exchange mechanisms involving functional groups present on the HAp@CT surface. Similar kinetic behavior has been reported for comparable multifunctional nanocomposites^49,50^.

**Table 3:** Kinetic parameters for the adsorption of Mn^2+^, Fe^3+^, and Pb^2+^ ions onto HAp, CT, and HAp@CT nanocomposite.

Kinetic models	Equations	Kinetic parameters	HAp			CT			HAp@CT		
Pseudo-First Order	Ln (q_e_−q_t_ ) = Ln (q_e_) − k_1_t	q_e_(mg g^− 1^)	Mn^2+^	Fe^3+^	Pb^2+^	Mn^2+^	Fe^3+^	Pb^2+^	Mn^2+^	Fe^3+^	Pb^2+^
			50.32	51.00	75.03	52.92	52.00	66.38	51.21	50.00	55.14
		K_1_ (min^− 1^)	0.0384	0.040	0.042	0.0336	0.035	0.0390	0.345	0.038	0.033
		R^2^	0.9664	0.980	0.96	0.9631	0.985	0.98	0.967	0.9888	0.99
Pseudo-Second Order	t/q_t_= 1/k_2_q_e_^2^+ t/q_e_	q_e_(mg g^− 1^)	47.78	48.05	62.80	48.42	47.80	131.43	48.29	48.20	40.87
		K_2_ (g mg^−1^min^− 1^)	0.0019	0.0020	6.7 × 10⁻⁵	0.0016	0.00016	3.9 × 10⁻⁵	0.0018	0.0018	3.0 × 10⁻⁶
		R^2^	0.9999	0.999	0.258	0.9997	0.999	0.735	0.9998	0.999	0.012
Intraparticle Diffusion	q_t_ = k_id_ t^1/2^ + C	K_id_ (mg g^−1^min^− 1/2^)	2.37	0.252	5.82	0.208	0.207	5.50	0.205	0.205	5.34
		C	43.76	43.20	−14.82	44.20	43.72	−10.40	44.15	43.91	−6.73
		R^2^	0.9983	0.9984	0.979	0.9993	0.9997	0.985	0.9987	0.9988	0.955

#### Effect of HAp@CT nanosorbent dosage

**Fig. 12:**
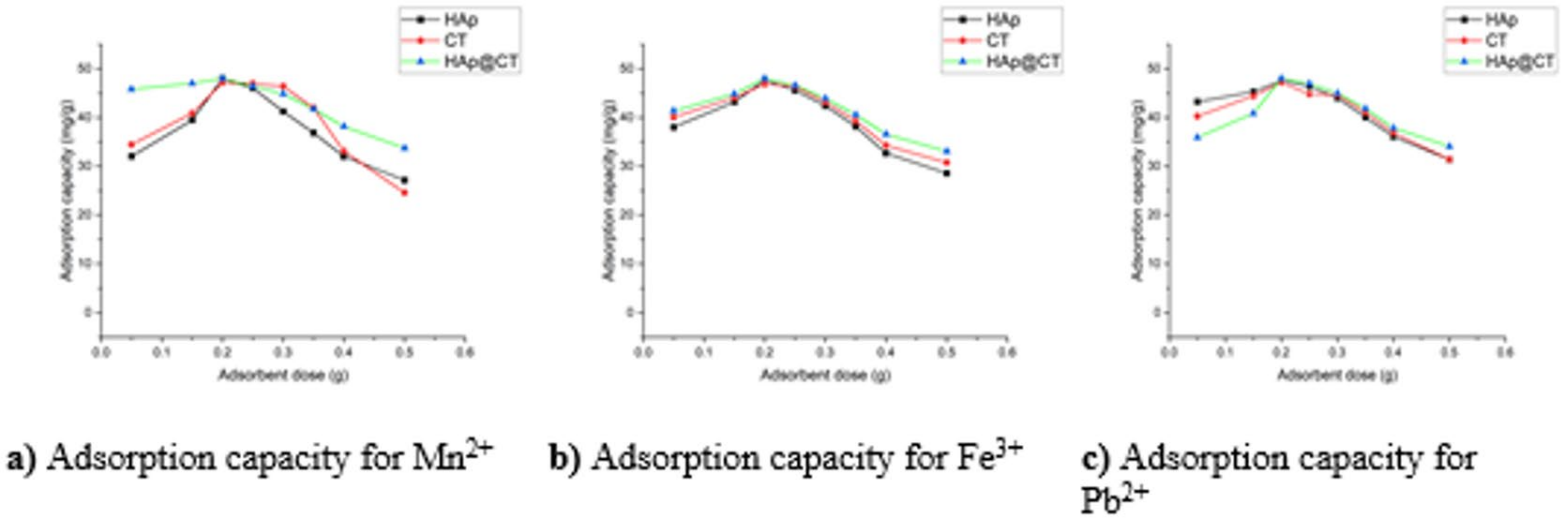
(**a–c**) Effect of Mass of nanosorbent (g) on the adsorption capacity HAp, CT, and HAp@CT for Mn^2+^, Fe^3+^, and Pb^2+^ respectively.

The effect of HAp@CT dosage on the removal efficiency of Mn^2+^, Fe^3+^, and Pb^2+^ ions was evaluated using adsorbent amounts ranging from 5.0 to 50.0 mg, as shown in Fig. 12a–c. All experiments were conducted under optimal conditions (pH 5.0–6.0 and a contact time of 120.0 min) for each metal ion using the three adsorbents. The adsorption capacity of all three nanomaterials decreased with increasing adsorbent dose. This behavior is attributed to the increase in the number of available binding sites relative to the fixed concentration of metal ions in solution^51,52^. At higher adsorbent dosages, particle aggregation may also occur, which can reduce the effective surface area available for adsorption. The highest adsorption capacities were observed at the lowest adsorbent dosage, highlighting the effective synergistic behavior of the HAp@CT nanocomposite, likely due to its enhanced surface area and improved accessibility of active adsorption sites for Mn^2+^, Fe^3+^, and Pb^2+^ ions. CT exhibited slightly lower performance, benefiting from its active amine (–NH_2_) and hydroxyl (–OH) functional groups, while pure hydroxyapatite showed comparatively lower adsorption capacity.

Overall, the removal efficiency followed the order: HAp@CT > CT > HAp. This result confirms that the HAp@CT nanocomposite is the most effective material for the selective removal of Mn^2+^, Fe^3+^, and Pb^2+^ ions from aqueous solutions^51,52^.

#### Effect of temperature

**Fig. 13:**
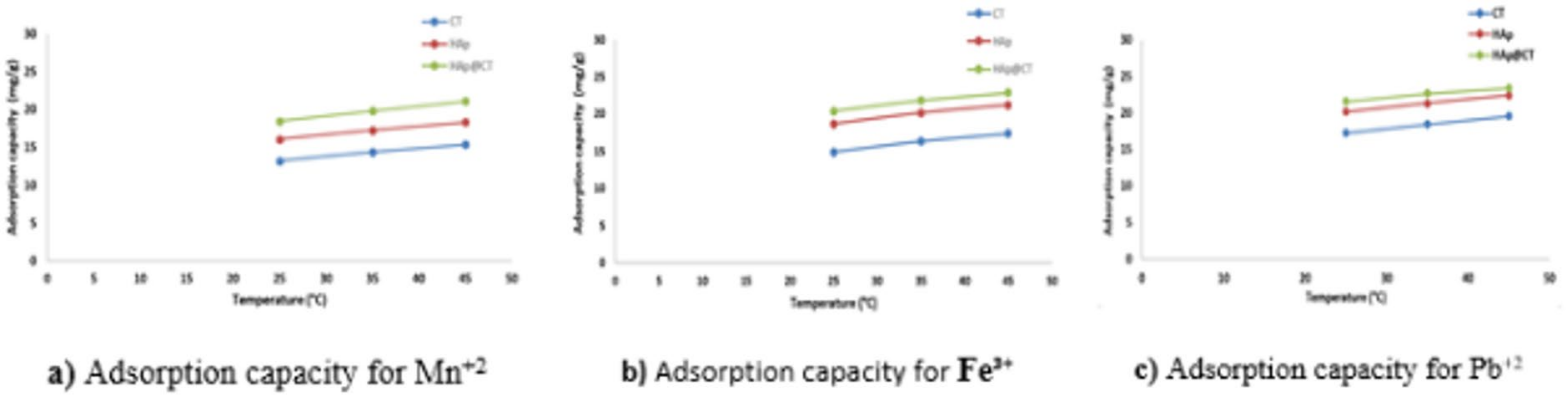
(**a–c**) Effect of temperatures (c) on the adsorption capacity HAp, CT, and HAp@CT for Mn^2+^, Fe^3+^, and Pb^2+^ respectively.

**Fig. 14:**
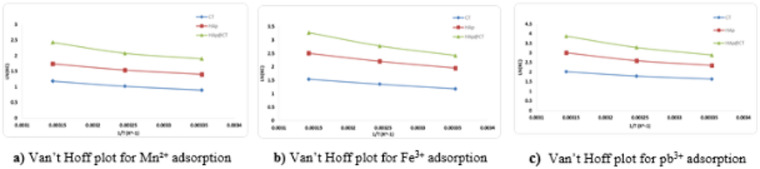
Van’t Hoff plots (ln Kc vs. 1/T) for the adsorption of Mn^2+^, Fe^3+^, and Pb^2+^ onto HAp, CT, and HAp@CT, illustrating the thermodynamic behavior of the adsorption process.

The effect of temperature on the adsorption behavior of HAp, CT, and HAp@CT toward Mn^2+^, Fe^3+^, and Pb^2+^ ions was investigated over 25–45 °C as illustrated in Fig. 13a–c. The adsorption capacities of all adsorbents increased gradually with temperature, indicating that higher thermal energy enhances metal ion interaction with available active sites and promotes diffusion toward internal adsorption sites^53,54^. This thermally favorable behavior is characteristic of endothermic adsorption and agrees with the positive ΔH° values presented in Table 6. Among the investigated adsorbents, HAp@CT consistently exhibited the highest adsorption capacity due to the synergistic combination of HAp and CT, which provides a greater density of functional groups and accessible binding sites for metal ion uptake^55^. These results demonstrate that HAp@CT maintains enhanced adsorption efficiency under varying thermal conditions, highlighting its potential for wastewater treatment applications. Thermodynamic parameters were evaluated at 25, 35, and 45 °C using ΔG° = −RT lnKc and the Van’t Hoff equation, lnKc = −ΔH°/RT + ΔS°/R^53,54^. The Van’t Hoff plots in Fig. 14 showed good linearity, confirming the applicability of the thermodynamic model, while the calculated parameters are summarized in Table 6. The negative ΔG° values, which became more negative with increasing temperature, indicate that adsorption is spontaneous and increasingly favorable at higher temperatures. The positive ΔH° values confirm the endothermic nature of the process, consistent with the observed increase in adsorption capacity, while the positive ΔS° values indicate increased randomness at the solid–solution interface due to the release of hydration water molecules during adsorption^53,54^. Furthermore, the ΔH° values suggest that adsorption on pristine HAp and CT is predominantly physical, whereas HAp@CT follows a mixed mechanism involving both physical adsorption and surface complexation, with stronger interactions toward Fe^3+^, and Pb^2+^ ions. Overall, HAp@CT exhibited the most favorable thermodynamic parameters, including more negative ΔG° and higher ΔS° values, confirming that the synergistic interaction between HAp and CT enhances adsorption affinity, active site accessibility, and the spontaneous, endothermic removal of Mn^2+^, Fe^3+^, and Pb^2+^ from aqueous solutions.

**Table 6:** Thermodynamic parameters for adsorption of Mn^2+^, Fe^3+^ and Pb^2+^ onto CT, HAp and HAp@CT.

Metal ion	Adsorbent	ΔH° (kJ mol^−1^)	ΔS° (J mol^−1^ K^−1^)	ΔG° (298 K)	ΔG° (308 K)	ΔG° (318 K)
Mn^2+^	CT	11.37	45.49	-2.22	-2.60	-3.13
Mn^2+^	HAp	13.29	56.13	-3.47	-3.95	-4.60
Mn^2+^	HAp@CT	20.67	84.89	-4.71	-5.33	-6.42
Fe^3+^	CT	14.10	57.09	-2.93	-3.48	-4.07
Fe^3+^	HAp	21.64	88.79	-4.86	-5.66	-6.64
Fe^3+^	HAp@CT	33.53	132.41	-6.02	-7.13	-8.68
Pb^2+^	CT	14.97	63.70	-4.06	-4.59	-5.34
Pb^2+^	HAp	25.76	105.70	-5.83	-6.65	-7.95
Pb^2+^	HAp@CT	38.58	153.10	-7.16	-8.40	-10.24

### Regeneration performance of the adsorbents

The regeneration performance of HAp, CT, and the HAp@CT composite was evaluated over five consecutive adsorption–desorption cycles using real industrial wastewater samples to assess the practical applicability of the prepared adsorbent and the results were tabulated in Table 5, the wastewater was collected from the backwashing process of iron–manganese removal filters at a groundwater treatment facility. The wastewater naturally contained low concentrations of Mn^2+^, Fe^3+^ and Pb^2+^ was spiked to achieve the desired initial concentration. In addition to Mn^2+^, Fe^3+^, the wastewater matrix contained typical coexisting ions, including Ca^2+^, Mg^2+^, Na^+^, K^+^, Cl^−^, and SO_4_^2−^. The pH of the wastewater ranged from 6.5 to 7.4, allowing evaluation of the adsorbent performance under more realistic conditions. For consistency with the adsorption experiments, each cycle was conducted using 100.0 mL of solution with the corresponding initial metal-ion concentration under continuous shaking conditions. After each adsorption cycle, the spent adsorbent was separated by centrifugation at 4000 rpm for 10.0 min and washed thoroughly with distilled water to remove loosely bound metal ions. Desorption was carried out using 0.1 M HCl solution under continuous shaking at 150 rpm for 60.0 min at room temperature. The regenerated material was then washed repeatedly with distilled water until neutral pH was reached and dried at 60.0 °C for 12.0 h before reuse in the subsequent cycle, following common regeneration procedures reported for heavy-metal adsorbents^57,]^.

**Table 5:** a, b, and c Remediation of Mn^2+^, Fe^3+^, and Pb^2+^ using HAp, CT, and HAp@ CT from real industrial wastewater.

Cycle	HAp	CT	HAp@ CT
(a) Remediation of Mn^2+^			
1	90.0%	92.5%	91.5%
2	88.5%	91.0%	90.2%
3	87.2%	88.0%	88.5%
4	85.9%	86.6%	86.8%
5	85.0%	85.2%	85.5%
(b) Remediation of Fe^3+^			
1	90.3%	91.5%	95.0%
2	88.6%	90.2%	92.5%
3	87.2%	89.5%	91.0%
4	85.9%	86.8%	88.6%
5	85.0%	85.1%	84.2%
(c) Remediation of Pb^2+^			
1	90.3%	95.0%	91.5%
2	88.6%	92.5%	90.2%
3	87.2%	91.0%	88.5%
4	85.9%	89.6%	86.8%
5	85.0%	88.2%	85.1%

For Mn^2+^ removal, all adsorbents exhibited high initial efficiencies exceeding 90%. A gradual decrease in removal efficiency was observed over successive cycles. After five cycles, HAp@CT retained 85.5% removal efficiency, which was comparable to HAp (85.0%) and CT (85.2%), indicating good structural stability during repeated use.

In the case of Fe^3+^ removal, HAp@CT demonstrated the highest initial efficiency (95.0%), followed by CT (91.5%) and HAp (90.3%). Although a moderate decline occurred across cycles, the composite maintained stable performance and slightly outperformed HAp in later cycles. Similarly, for Pb^2+^ removal, high initial efficiencies were recorded for all materials. After five regeneration cycles, the adsorption efficiency remained above 85% for each adsorbent, confirming their reusability and chemical stability under the applied regeneration conditions.

Overall, while CT showed slightly higher initial removal efficiencies for certain metal ions, the HAp@CT composite demonstrated balanced adsorption capacity and consistent regeneration behavior, highlighting its potential as a reusable adsorbent for industrial wastewater treatment applications^57,58^.

### Comparative performance with previously reported adsorbents

The adsorption performance of the synthesized HAp@CT composite was compared with various reported adsorbents for heavy-metal removal, as summarized in Table 4. Although some adsorbents reported in the literature exhibit higher adsorption capacities, the developed composite provides several practical advantages. The material is synthesized from low-cost shrimp-waste–derived chitosan through a simple and environmentally friendly process. In addition, the composite demonstrates effective adsorption toward multiple metal ions and maintains stable performance during repeated regeneration cycles. These characteristics highlight the potential of the HAp@CT composite as a sustainable and reusable adsorbent for wastewater treatment applications.

**Table 4:** Comparison of the adsorption performance of the synthesized HAp@CT composite with previously reported adsorbents for heavy-metal removal.

Adsorbent/composite type	Composite components	Target heavy metal(s)/pollutant(s)	Adsorption capacity, qmax (mg/g)	Optimum pH	Contact time (min)	References
HAp@CT composite (this study)	Hydroxyapatite/chitosan composite	Mn^2+^, Fe^3+^, Pb^2+^	229.02 (Mn^2+^), 206.92 (Fe^3+^), 179.33 (Pb^2+^)	5–6	120	This work
Low-cost adsorbents	Agricultural and industrial waste-derived adsorbents	Pb^2+^, Cd^2+^, Ni^2+^	20–300	4–7	30–240	^1^
Ag nanoparticles/magnetic nanocomposites	Silver nanoparticles + magnetic oxides	Ni^2+^, Cu^2+^, Pb^2+^, Cd^2+^	70–180	5–7	30–120	^4^
Nanomaterial-enhanced membranes	Polymeric membranes incorporated with nanomaterials	Pb^2+^, Cd^2+^, Cr⁶⁺ and other heavy metals	60–250	5–8	Continuous flow	^5^
Hydroxyapatite adsorbents	Pure or modified hydroxyapatite	Pb^2+^, Cd^2+^, Cu^2+^, Zn^2+^	80–300	4–7	20–240	^7^
Hydroxyapatite-based adsorbents	Hydroxyapatite composites and nanostructures	Pb^2+^, Cd^2+^, Cu^2+^, Zn^2+^, Ni^2+^	50–350	4–7	30–240	^8^
Hydroxyapatite composites	HAp combined with polymers or inorganic fillers	Pb^2+^, Cd^2+^, Cu^2+^, Cr^3+^	60–320	5–7	30–180	^9^
Hydroxyapatite nanocomposites	Nano-HAp incorporated into hybrid matrices	Pb^2+^, Cd^2+^, Ni^2+^, Hg^2+^	100–350	4–7	30–180	^10^
Chitosan–hydroxyapatite composite	Chitosan/HAp photocatalyst	Acid Orange 7 dye	~ 65	6–7	60	^11^
Chitosan–hydroxyapatite biofilm composite	Chitosan/HAp film-form biocomposite	Pb^2+^, Cu^2+^, Cd^2+^	70–150	5–7	60–180	^12^
Mn-ferrite/HAp nanocomposite	Manganese ferrite + hydroxyapatite	Pb^2+^, Cd^2+^, Cu^2+^	120–280	5–7	30–120	^13^
Chitosan–collagen/HAp nanocomposite	Chitosan + collagen + hydroxyapatite	Cu^2+^	~ 140	5.5	90	^14^
Chitosan/nano-HAp composite	Chitosan + nano-hydroxyapatite	Cd^2+^	~ 135	6.0	120	^15^
Magnetic hydroxyapatite nanocomposite	Magnetic particles + hydroxyapatite	Pb^2+^, Hg^2+^, As^3+^	150–320	5–6	60	^16^
Hydroxyapatite/chitosan composites	HAp/chitosan hybrid adsorbents	Pb^2+^, Cd^2+^, Cu^2+^, Zn^2+^	80–260	5–7	60–180	^17^
Chitosan-based beads	Crosslinked chitosan biopolymer beads	Pb^2+^, Cu^2+^, Cd^2+^, dyes	70–200	4–7	30–180	^18^
AgNPs/GO/chitosan composite	Silver nanoparticles + graphene oxide + chitosan	Mn^2+^	~ 160	6.0	60	^19^
Magnetic chitosan nanocomposites	Chitosan with magnetic nanoparticles	Cr^3+^, Fe^3+^, Mn^2+^	100–240	4–6	60–120	^20^
Heavy-metal adsorbents (reviewed nanomaterials)	Various nano-adsorbents	Pb^2+^, Cd^2+^, Cu^2+^, Cr⁶⁺, Ni^2+^	40–350	3–8	10–240	^21^
Nanocomposite adsorbents	Organic/inorganic nanocomposites	Pb^2+^, Cd^2+^, Cr⁶⁺, dyes	50–320	4–7	20–240	^22^
Chitosan-based adsorbents	Functionalized chitosan materials	Pb^2+^, Cd^2+^, Cu^2+^, Ni^2+^	50–300	4–7	30–240	^23^
Nano-HAp/chitosan biocomposite from fish and shrimp waste	Waste-derived nano-HAp/chitosan	Pb^2+^, Cd^2+^, Cu^2+^	100–260	5–7	60–180	^24^
Porous hydroxyapatite sheets	Porous structured HAp sheets	Pb^2+^	~ 190	5.5	120	^25^
HAp–cellulose nanofibril biosorbent	Hydroxyapatite + cellulose nanofibrils	Cr(VI), Pb^2+^	90–220	4–6	60–180	^43^
Hydroxyapatite–chitosan remediation composites	HAp/chitosan environmental composites	Pb^2+^, Cd^2+^, Cu^2+^, dyes	80–300	4–7	30–180	^44^
Mn-doped hydroxyapatite	Manganese-substituted hydroxyapatite	Pb^2+^, Cd^2+^	150–290	5–6	60–120	^45^
Chitosan/fluorapatite composite	Chitosan + fluorapatite	Pb^2+^	~ 170	5–6	60–120	^46^

## Conclusion

In this study, hydroxyapatite (HAp), chitosan (CT), and their composite material (HAp@CT) were successfully synthesized using shrimp waste-derived CT as a sustainable precursor for the preparation of an efficient adsorbent for heavy metal removal from aqueous solutions. Comprehensive characterization techniques including FT-IR, XRD, TGA, TEM, SEM, EDX, and zeta potential analysis confirmed the successful formation of the HAp@CT nanocomposite and demonstrated its structural stability and favorable surface properties. Batch adsorption experiments revealed that the HAp@CT nanocomposite exhibited superior adsorption performance toward Mn^2+^, Fe^3+^, and Pb^2+^ ions compared with the individual HAp, and CT materials. The enhanced adsorption efficiency can be attributed to the synergistic interaction between HAp and CT, which provides abundant active sites including phosphate, hydroxyl, and amine functional groups. Kinetic studies indicated that the adsorption process followed the pseudo-second-order model, suggesting that chemisorption plays a significant role in the adsorption mechanism. Thermodynamic analysis demonstrated that the adsorption process is spontaneous and endothermic, while regeneration experiments confirmed that the adsorbent maintains high removal efficiency after multiple reuse cycles. Overall, the results demonstrate that the HAp@CT nanocomposite represents a promising, environmentally friendly, and cost-effective material for the removal of heavy metals from contaminated water and industrial wastewater.

## Data Availability

All data generated or analyzed during this study are included in this published article. Additional data may be available from the corresponding author upon reasonable request.
